# Navigating MASLD heterogeneity: from metabolic risk to precision stratification

**DOI:** 10.3389/fmed.2026.1966460

**Published:** 2026-10-30

**Authors:** Chenyue Yan, Guixiang Zhang, Libo Liang, He He

**Affiliations:** 1 Department of Laboratory Medicine, West China Hospital, Sichuan University, Chengdu, Sichuan, China; 2 Gastric Cancer Center, West China Hospital, Sichuan University, Chengdu, Sichuan, China; 3 Division of Gastrointestinal Surgery, Department of General Surgery, West China Hospital, Sichuan University, Chengdu, Sichuan, China; 4 General Practice Medical Center, West China Hospital, Sichuan University, Chengdu, Sichuan, China; 5 Sichuan Clinical Research Center for Laboratory Medicine, West China Hospital, Sichuan University, Chengdu, Sichuan, China; 6 Clinical Laboratory Medicine Research Center, West China Hospital, Sichuan University, Chengdu, Sichuan, China

**Keywords:** heterogeneity, metabolic dysfunction-associated steatohepatitis, metabolic dysfunction-associated steatotic liver disease, non-invasive biomarkers, personalized therapeutics, precision medicine, risk stratification, translational medicine

## Abstract

Metabolic dysfunction-associated steatotic liver disease (MASLD) is a heterogeneous multisystem condition characterized by hepatic steatosis occurring in the context of metabolic dysfunction, and its prevalence continues to rise globally in parallel with the epidemics of obesity, type 2 diabetes, and metabolic syndrome. The heterogeneity of MASLD spanning etiology, pathophysiology, clinical presentation, and therapeutic response, poses substantial challenges for clinical management and underscores the need for a stratified approach to diagnosis and treatment. This review provides a comprehensive overview of MASLD heterogeneity, tracing the evolution of its classification from the exclusion-based NAFLD/NASH paradigm to the current positive-diagnosis MASLD framework with four principal subtypes: isolated MASLD, MetALD, MASH, and MASLD with other liver diseases. The multifactorial etiology of MASLD is examined, encompassing hepatic lobular spatial heterogeneity, lipid overload and inflammatory pathways, genetic and epigenetic determinants, and modifiable environmental risk factors. The heterogeneous clinical manifestations, including the distinct phenotypes of lean vs. obese MASLD and variable natural disease trajectories, are discussed. Diagnostic strategies are evaluated across the spectrum of disease severity, from non-invasive screening tools (MRI-PDFF, CAP, FIB-4, elastography) to histological assessment of MASH. Therapeutic approaches, including lifestyle modification, pharmacological therapies, and surgical interventions, are reviewed. Recognizing and systematically addressing this heterogeneity is essential for advancing precision medicine in this field. Based on subtype classification, risk factor profiling, and stage-appropriate diagnostic and therapeutic strategies, this review presents a stratification framework that offers clinicians a practical roadmap for delivering more personalized and effective care across the diverse spectrum of MASLD patients. In contrast to recent comprehensive reviews that provide broad overviews of MASLD, this review specifically addresses how disease heterogeneity should drive clinical decision-making, bridging the gap between subtype classification and personalized therapeutic selection.

## 1 Introduction

Metabolic dysfunction-associated steatotic liver disease (MASLD) is characterized by hepatic steatosis occurring in the context of metabolic dysfunction, with particular emphasis on the latter (1). The prevalence of MASLD has risen substantially, driven by the increasing rates of type2 diabetes mellitus (T2DM), obesity, and metabolic syndrome (2–5). Notably, several studies have reported a higher prevalence of MASLD and more pronounced phenotypic manifestations in men than in women in certain populations (6, 7). The estimated global prevalence of MASLD is approximately 31% among adults, with the majority of cases presenting at an early stage as isolated steatosis. The highest prevalence rates are observed in the Middle East (43%) and Latin America (35%), while the lowest rates are found in Central Africa and South Africa (13%) (8–10). According to data available as of 2024, MASLD now affects over 25% of the global population and is the leading cause of chronic liver disease in China, accounting for 50% of cases (10, 11).

The term MASLD underscores its strong association with various metabolic disorders such as insulin resistance, obesity, hypertension(HTN), and hyperlipidemia. This broader definition encompasses liver fat deposition from multiple causes, rather than focusing solely on the effects of alcohol (12). The new nomenclature highlights the heterogeneity of the disease in pathology and clinical manifestations, providing researchers and clinicians with a framework to develop more effective treatment options for different disease stages based on MASLD classification (12). In addition, this nomenclature reflects the influence of genetic and environmental factors on disease pathogenesis. Substantial variation among patients in genetic background, lifestyle habits, diet, and physical activity, may contribute to the heterogeneity of clinical presentation (13, 14).

Therefore, this article reviews the heterogeneity of MASLD in terms of its subtypes, etiology, risk factors, clinical manifestations, diagnostic methods, and therapeutic strategies. It specifically addresses heterogeneity in clinical presentations, diagnostic approaches, and therapeutic interventions, with the aim of advancing stratified management strategies for MASLD through improved clinical precision in risk prediction, prevention, and treatment.

While several landmark reviews have recently provided excellent comprehensive overviews of MASLD (13), these reviews have primarily focused on the pathogenesis, clinical outcomes, and metabolic determinants of treatment response heterogeneity in MASLD. However, comprehensive reviews addressing genetic and environmental risk factors, as well as diagnostic strategies, remain limited. The present review fills this gap by proposing a stratification framework that directly links subtype classification, genetic and environmental risk profiling, and stage-appropriate diagnostic-therapeutic coupling to individualized patient management. This translational focus, from heterogeneity characterization to precision stratification, distinguishes the current work from existing literature and provides clinicians with actionable decision tools rather than descriptive summaries.

## 2 Methodology

This narrative review synthesizes evidence from articles selected over the past decade that are relevant to the clinical management of MASLD, with an emphasis on recent publications from 2020 to 2026. We conducted a targeted search of the following electronic databases: PubMed and Embase. last search date was June 30, 2026. Duplicate articles retrieved from both databases were included only once in the review process. The search strategy combined MeSH terms and free-text keywords, including (MASLD OR NAFLD OR MASH OR NASH OR metabolic dysfunction-associated steatotic liver disease OR metabolic dysfunction-associated steatohepatitis OR nonalcoholic fatty liver disease OR nonalcoholic steatohepatitis OR MetALD) AND (heterogeneity OR subtype OR etiology OR diagnosis OR manifestation OR management OR treatment OR lifestyle intervention OR pharmacotherapy OR surgery OR gene OR environment OR risk factor), limited to English-language publications. To ensure the comprehensiveness of the data, we also reviewed the literature cited in previous meta-analyses, key reviews, and clinical practice guidelines on MALSD to identify any relevant studies that might have been overlooked.

Studies were included if they addressed any aspect of MASLD epidemiology, pathophysiology, diagnosis, or therapeutic strategies, with priority given to meta-analyses, systematic reviews, randomized controlled trials, and major clinical practice guidelines. Editorials, case reports, conference abstracts, and non-English articles were excluded. Two independent reviewers (C. Yan and G. Zhang) initially screened titles and abstracts, followed by full-text assessment against predefined inclusion criteria. Discrepancies were resolved through discussion or consultation with a third reviewer (H. He). Of the 12,391 records initially identified through the database search, 224 studies met the predefined inclusion criteria and were ultimately included in this review. The final evidence base comprised 114 observational studies (50.9%), 13 randomized controlled trials (5.8%), 35 systematic reviews or meta-analyses (15.6%), 50 narrative reviews(22.3%), and 12 clinical guidelines or expert consensus statements (5.4%). The distribution of documentary evidence is illustrated in the Evidence Landscape diagram (Supplementary Figure S1), and the quality of core included studies is summarized in the Evidence Quality Assessment (Supplementary Table S1).

## 3 The historical development of MASLD subtype classification

The conceptual framework and classification system for steatotic liver disease (SLD) have undergone a significant evolution since the 1980s. This evolution is characterized by a shift from a historically exclusion-based, negatively defined diagnostic approach toward a contemporary, criteria-based positive diagnostic paradigm, culminating in the recent establishment of a detailed stratification system designed to capture the condition's clinical heterogeneity.

From approximately 1980 to 2020, the field was dominated by the non-alcoholic fatty liver disease (NAFLD)/non-alcoholic steatohepatitis (NASH) paradigm (15, 16). Under this framework, the NAFLD and its progressive subtype, NASH, relied fundamentally on an exclusion-based approach. This required confirming hepatic steatosis and then excluding significant alcohol consumption and other known secondary causes. The framework broadly categorized the disease into “simple fatty liver” and biopsy-confirmed NASH (17). Key limitations of this paradigm included the stigmatizing and negatively defined nature of the term “fatty,” the practical challenges of applying exclusion criteria in patients with multiple comorbidities, and its inability to adequately address cases where metabolic risk factors coexisted with alcohol use or other liver diseases (12, 18, 19).

A fundamental transformation began in 2020 with the proposal of metabolic associated fatty liver disease (MAFLD), a concept that remained central until 2023 (20, 21). The MAFLD paradigm introduced a positive diagnostic criterion: the presence of hepatic steatosis plus at least one of the following: overweight or obesity, type 2 diabetes, or two other specified metabolic risk factors (19). This approach explicitly acknowledged that MAFLD could coexist with other liver diseases, thereby shifting the diagnostic focus from “excluding other causes” to “confirming the disease itself” and paving the way for more precise subtyping (19).

Since 2023, international consensus efforts led by major hepatology societies, including the European Association for the Study of the Liver (EASL) and the American Association for the Study of Liver Diseases (AASLD), have further refined the terminology and classification (22, 23). Building on the positive-diagnosis principle of MAFLD, the nomenclature has been updated to MASLD to more accurately reflect its pathophysiological basis (12, 24). The diagnostic criteria now employ a globally consensus-driven definition of cardiometabolic risk factors. Crucially, the new framework incorporates alcohol intake as a ke*y* axis for subtyping, using defined thresholds (≥30 g/day for men, ≥20 g/day for women) (22, 23). The classification of MASLD is illustrated in Figure 1. The nomenclature employed across studies may vary, but this review utilizes the most recent 2023 MASLD nomenclature. Subtype descriptions from the included literature were systematically compared and cross-validated to ensure accurate classification into the appropriate MASLD subtypes.

**Figure 1:**
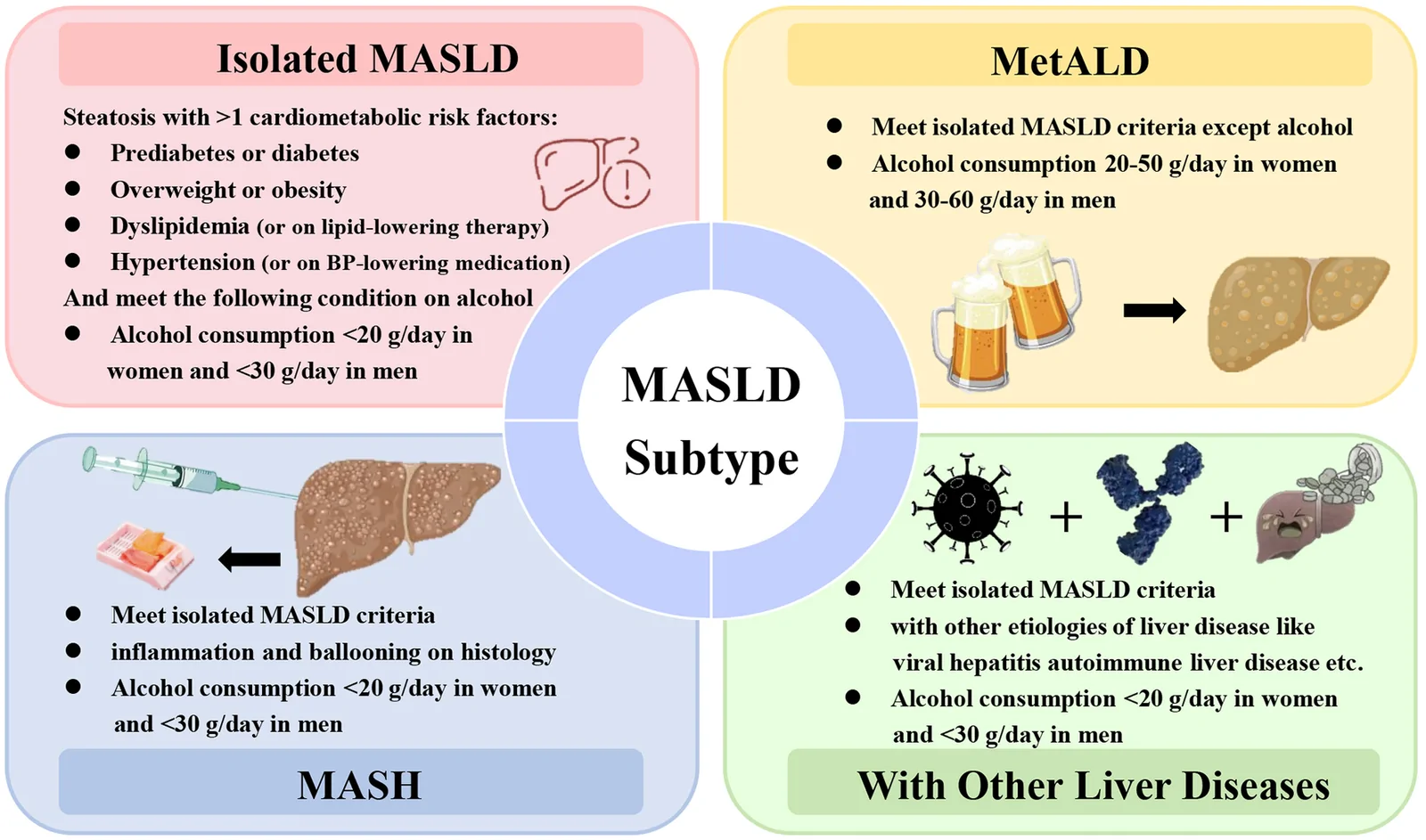
Schematic overview of the update metabolic dysfunction-associated steatotic liver disease (MASLD) subtypes. Isolated MASLD, Metabolic and Alcohol-Related Liver Disease (MetALD), Metabolic Dysfunction-Associated Steatohepatitis (MASH), and MASLD with Other Etiologies.

## 4 Etiology of MASLD

The pathogenesis of MASLD is rooted in systemic metabolic dysfunction, with insulin resistance serving as a central driver. Epidemiological data consistently link MASLD with obesity and type 2 diabetes (25), and even lean individuals with MASLD exhibit insulin resistance (26). At the molecular level, free fatty acid (FFA) accumulation is the hallmark of early MASLD (27). The liver obtains FFAs through three principal pathways: (1) Uptake of FFAs from the circulation. Excessive mobilization of FFAs produced by adipose tissue lipolysis, driven by insulin resistance, is the main source of liver triglycerides (TGs) (approximately 60%) (28). (2) *de novo* lipogenesis (DNL) accounts for approximately 26% of intrahepatic TGs. Excess carbohydrates are converted into FFAs in the liver through the DNL process. This process is strictly regulated by transcription factors (TFs), the most important of which is sterol regulatory element-binding protein-1c (SREBP-1c). SREBP-1c promotes DNL by upregulating the transcription of lipogenic genes such as acetyl-CoA carboxylase (ACC), fatty acid synthase (FAS) and stearoyl-CoA desaturase (SCDs). Hyperinsulinemia also causes upregulation of SREBP-1c, thereby promoting the DNL process and creating a vicious cycle of lipid overload (29).(3) Dietary fat accounts for approximately 15% of intrahepatic TGs. Saturated fatty acids are more lipotoxic than unsaturated fatty acids (including monounsaturated fatty acids and polyunsaturated fatty acids). Palmitic acid and stearic acid are the principal accumulated saturated fatty acids and are associated with the progression of MASLD (30).

Under physiological conditions, FFAs are disposed of through mitochondrial *β*-oxidation or re-esterification into triglycerides for VLDL secretion (31). When FFA uptake and DNL exceed the capacity of *β*-oxidation and VLDL secretion, triglyceride homeostasis is disrupted, leading to steatosis, through this alone does not necessarily lead to hepatocellular injury of fibrosis (32). However, when lipids accumulate excessively, reactive oxygen species (ROS) and lipotoxic substances, such as lysphosphatidylcholines (LPCs), diacylglycerols (DAGs), and ceramides, are generated, causing lipotoxicity. Lipid accumulation also induces oxidative stress, endoplasmic reticulum stress, and inflammasome activation, recruiting pro-inflammatory cells whose reciprocal interactions amplify and sustain the inflammatory response, thereby promoting the progression of MASLD (Figure 2) (27). This is a critical step in the transition from simple steatosis to steatohepatitis. Importantly, lipotoxicity and inflammatory pathways may also contribute to the pathogenesis of MASLD in lean individuals (33), highlighting the importance of metabolic dysfunction, visceral adiposity (34), and other pathogenic factors beyond overall adiposity (35).

**Figure 2:**
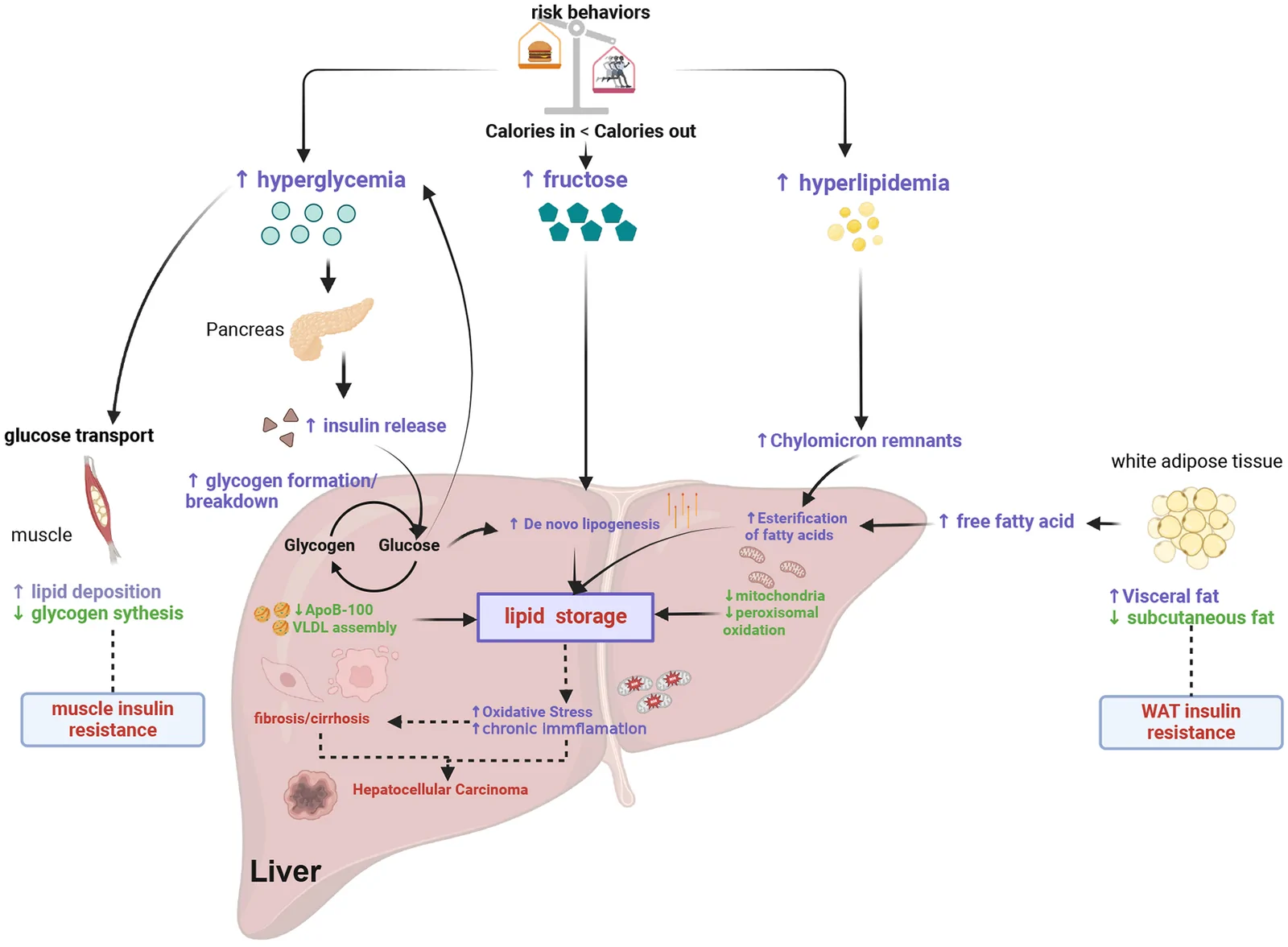
Mechanisms of lipid overload and inflammatory response in MASLD. Adverse cardiometabolic risk behaviors drive hepatic lipid accumulation via three parallel pathways: **(a)** hyperglycemia-induced insulin resistance stimulating hepatic glycogen formation and *de novo* lipogenesis, exacerbated by skeletal muscle insulin resistance releasing free fatty acids; **(b)** excessive fructose consumption directly fueling hepatic *de novo* lipogenesis; **(c)** hyperlipidemia, chylomicron remnants, and free fatty acids from visceral adipose tissue increasing hepatocyte lipid burden via esterification. Sustained lipid overload triggers mitochondrial/peroxisomal dysfunction, oxidative stress, and chronic inflammation, promoting fibrosis and cirrhosis that predispose to hepatocellular carcinoma.

Importantly, this transition is not uniform across the liver parenchyma. The severity of injury is influenced by the spatial heterogeneity of the hepatic lobule (36). Hepatocytes along the central axis exhibit differential gene expression, metabolic capacity, and susceptibility to damage, which may explain the zonal distribution of steatosis and fibrosis observed in MASLD (37). Emerging evidence highlights the spatial heterogeneity of hepatic lipid metabolism and steatosis in MASLD. Experimental studies have demonstrated distinct roles of insulin signaling in regulating glucose and lipid metabolism across periportal and pericentral hepatocytes (38). Furthermore, lipid accumulation has been shown to preferentially occur in the pericentral region during early MASLD, with zonated liver sinusoidal endothelial cells contributing to the regulation of hepatic steatosis (39). Importantly, the heterogeneous distribution of histopathological lesions throughout the hepatic parenchyma may also affect the reliability of liver biopsy interpretation. Consequently, biopsy specimens obtained from different hepatic regions may yield discordant findings, particularly regarding lobular inflammation, hepatocellular ballooning, and fibrosis staging (40–43).

## 5 Risk factors in MASLD

MASLD exhibits substantial phenotypic diversity, encompassing subtypes that range from simple steatosis to steatohepatitis, and from isolated hepatic involvement to multisystemic complications. However, this clinical heterogeneity represents only the visible tip of a far more complex biological reality. Beneath it lies a multilayered interplay among genetic susceptibility, environmental exposures, and epigenetic modifiers (Figure 3).

**Figure 3:**
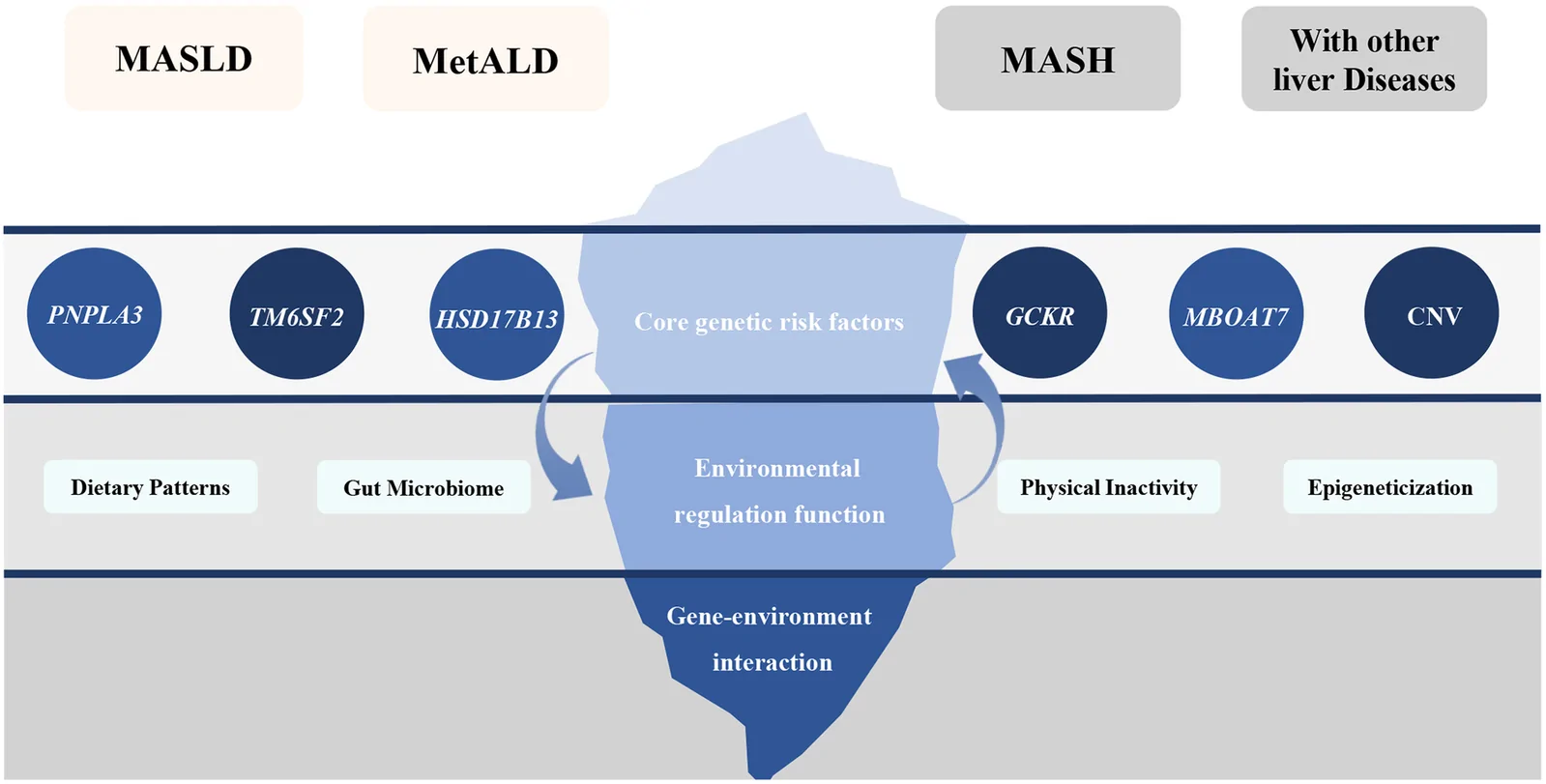
The iceberg of complexity from clinical phenotypes to genotypic and environmental determinants. The visible upper layer represents the diverse clinical phenotypes, encompassing Isolated MASLD, MetALD, MASH, and MASLD with other liver diseases. Beneath this surface lies a continuum of determinants: the middle layer highlights core genetic risk factors [PNPLA3, TM6SF2, HSD17B13, GCKR, MBOAT7, and Copy Number Variations (CNV)] alongside critical environmental regulatory functions, including dietary patterns, gut microbiome, physical inactivity, and epigenetic modifications. The submerged base signifies the profound and often hidden gene-environment interactions, which collectively drive disease pathogenesis and heterogeneity.

### 5.1 Genetic risk as a determinant of MASLD heterogeneity

Genome-wide association studies (GWAS) have identified multiple genetic loci associated with susceptibility to MASLD and its progression, including variants in *patatin-like phospholipase domain-containing protein 3* (*PNPLA3*), *transmembrane 6 superfamily member 2* (*TM6SF2*), *glucokinase regulator* (*GCKR*), *membrane-bound O-acyltransferase domain containing 7* (*MBOAT7*), and *17β-hydroxysteroid dehydrogenase type 13* (*HSD17B13*) (44). Notably, recent genetic evidence suggests that these variants contribute to MASLD through distinct biological pathways, including hepatocyte-intrinsic lipid handling, hepatic *de novo* lipogenesis, and systemic metabolic dysfunction, thereby contributing to heterogeneity in disease severity and clinical outcomes (45). Using data from the UK Biobank, Liu et al. (46) integrated five variants *PNPLA3* rs738409 C/G, *TM6SF2* rs58542926 C/T, *GCKR* rs1260326T/C, *MBOAT7* rs641738 C/T and *HSD17B13* rs72613567T/TA into the genetic risk score (GRS) for MAFLD. Their findings demonstrated that a higher GRS was associated with an increased risk of adverse hepatic outcomes, with variants in *PNPLA3*, *TM6SF2*, and *MBOAT7* making the predominant contributions.

PNPLA3 localizes to hepatic lipid droplets and can interfere with adipose triglyceride lipase activity. *PNPLA3* rs738409 C > G variant has been shown to be the strongest genetic variant associated with MASLD across all ethnic populations (47). Further cohort and case-control studies have delineated the interaction effects of metabolic and genetic risk factors on the progression of MASLD. *PNPLA3* variants increase the risk of MASLD but have no effect on fatty liver without metabolic dysfunction (48), implying that metabolic dysregulation is a prerequisite for PNPLA3-driven hepatic steatosis (49). In a study with a median follow-up of 94 months, liver and extrahepatic disease progression was not associated with *PNPLA3* genotype in lean MASLD patients of Caucasian ethnicity (50), underscoring a possible gene-adiposity interaction. Complementary GWAS work by Hsu et al. (51) found that the *PNPLA3* rs9625962 C allele is associated with a higher risk of MASLD across both lean and non-lean populations. Concurrently, patients harboring the *PNPLA3* I148M (rs738409) G/G risk genotype exhibit elevated levels of activator protein A, a factor potentially implicated in the progression from simple steatosis to hepatic fibrosis (52). The lean-Caucasian negative finding (50) and Hsu's lean-positive result (51) likely reflect differences in BMI cut-off values and ethnicity. This inconsistency itself argues for the use of GRS rather than single-variant approaches in lean MASLD. Meanwhile, the presence of *PNPLA3* risk alleles demonstrates an independent gene-dose effect, correlating with an escalating risk of significant fibrosis (53). Building on this intrinsic risk, *PNPLA3* may further interact with environmental exposures, collectively influencing disease susceptibility and progression. In the UK Biobank, Chen et al. (54) reported that the *PNPLA3* susceptibility genotype modifies the association between habitual salt addition and the risk of developing MASLD.

TM6SF2 is a membrane protein located in the endoplasmic reticulum (ER) and the ER-Golgi intermediate compartment. Silencing *TM6SF2* in mouse liver impairs the lipidation of apolipoprotein B (ApoB) lipoprotein, reduces the secretion of very low-density lipoprotein triglyceride (VLDL-TG) form the liver, and leads to hepatic steatosis (55). According to the exome-wide association study (EWAS) analysis in the multi-ethnic population of the Dallas Heart Study, *TM6SF2* rs58542926 C > T variant was associated with hepatic steatosis and decreased plasma cholesterol and triglycerides (TG) (56). In a multicenter study of Swedish Obese Subjects (SOS), although *TM6SF2* rs58542926 C > T promoted adverse intrahepatic events in MASLD, it was a protective factor for extrahepatic cardiovascular disease (CVD) (57). Similar to the *PNPLA3* variant, the *TM6SF2* variant promoted the progression of MASLD but had no effect on fatty liver disease without metabolic dysfunction (48).

MBOAT7, also known as lysophosphatidylinositol acyltransferase 1 (LPIAT1), encodes a protein with six transmembrane domains located in the endoplasmic reticulum (58). *MBOAT7* exhibits lysophosphatidic acyltransferase activity, which catalyzes the binding of arachidonic acid to phosphatidylinositol (PI) (59). The *MBOAT7* variant is considered to be an independent risk factor for MASLD progression (60). The relationship between *MBOAT7* rs641738 C>T and liver cirrhosis was first reported in GWAS analysis of alcohol abusers (61). A cohort study at the Dallas Heart Study Center showed that the *MBOAT7* rs641738 variant was associated with the progression and severity of MASLD by altering the remodeling of hepatic PI acyl chains (62). A meta-analysis of 40 studies by Teo et al. (63) confirmed that the *MBOAT7* rs641738 variant was associated with elevated serum ALT and decreased serum TG, but not with fasting insulin. Torre et al. (64) identified an association between *MBOAT7* variants and more severe hepatic steatosis, indicated by higher CAP values. Abdel Sattar et al. (65) reported a higher frequency of the *MBOAT7* rs641738T allele and the T/T genotype in MASLD patients with significant fibrosis. Therefore, *MBOAT7* is an independent risk factor for the progression of MASLD.

In contrast to risk-increasing variants in *PNPLA3* and *TM6SF2,* the *HSD17B13* rs72613567 splice variant has been associated with protection against progressive liver injury. *HSD17B13* encodes a hepatocyte-enriched, lipid droplet-associated enzyme, and the protective allele alters RNA splicing, resulting in reduced protein stability and altered enzymatic function (66). Importantly, this variant is associated with a lower risk of steatohepatitis and advanced fibrosis rather than a consistent reduction in hepatic steatosis (66). Mechanistic studies have linked *HSD17B13* inhibition to reduced hepatic pyrimidine catabolism, which attenuates liver fibrogenesis (67). More recently, experimental evidence demonstrated that *Hsd17b13* knockdown decreases hepatic lipid droplet cholesterol content and alleviates inflammatory and profibrotic responses in a mouse model of MASH, without significantly altering steatosis (68). Furthermore, a 2025 clinical study incorporated protective *HSD17B13* variants into genetic risk models associated with age-related increases in liver stiffness among patients with MASLD (69). Together, these findings highlight the role of *HSD17B13* in modifying liver disease progression and provide a rationale for investigating *HSD17B13*-targeted therapeutic strategies.

Beyond SNPs, copy number variation (CNV) is another common form of genetic alteration. Emerging evidence from **s**mall-scale genomic studies suggests that CNV can affect gene expression and is an important driver of disease development. CNV differs from SNP in size and mutation rate, but few studies have examined the correlation between CNV and MASLD (70). In a case-control study employing genome-wide CNV analysis, Zain et al. (71) identified four rare and/or novel CNV loci unique to MASLD (33q13.12, 11q21.11, 1p11.2-12.13 and 2q49.49). Another cohort study by Metwally et al. (72) found that the duplication of XPO4 copy number variation was associated with the severity of liver fibrosis. By reducing the level of XPO4 in the liver, SMAD3/SMAD4 is continuously activated, promoting TGF-*β*1-mediated activation and fibrosis of hepatic stellate cells.

While genetic background plays a critical role in MASLD pathogenesis and progression, accumulating evidence from experimental and translational studies also underscores the importance of epigenetics (73). Epigenetics refers to heritable phenomena that alter gene expression without changing the DNA sequence. Reversible epigenetic changes occur at the transcriptional level and provide a phenotypic link between the host and the environment. Beyond genetic predisposition, observational and mechanistic studies have demonstrated that unhealthy lifestyle factors such as overnutrition, physical inactivity and weight gain can influence the development and progression of MASLD through epigenetic mechanisms (74, 75). Recent studies suggest that epigenetic mechanisms constitute a flexible regulatory pathway connecting the host and the environment, and hold promise as therapeutic targets (76).

Three principal epigenetic regulatory mechanisms have been described: (1) DNA nucleotide modification (DNA methylation); (2) histone (modification histones are the main protein components of nucleosomes), which determines the density and accessibility of chromatin (histone tail modification); and (3) regulation of RNA processing, stability and translation through specific binding of small RNA molecules (77). Preclinical and clinical studies have implicated epigenetic modifications in liver lipid metabolism, insulin resistance, mitochondrial dysfunction, endoplasmic reticulum stress, and oxidative stress, all of which are closely related to the development and progression of MASLD (78). Given that patients with MASLD have disorders of glucose and lipid metabolism, epigenetic intervention through nutrition may contribute to the prevention and management of MASLD (79).

In conclusion, genetic factors are among the major determinants of MASLD susceptibility and progression. Genetic risk scores incorporating *PNPLA3*, *TM6SF2*, and *HSD17B13* variants are approaching clinical readiness. Patients carrying high-risk alleles may benefit from earlier screening, intensified lifestyle interventions, and prioritization for pharmacotherapy. The protective HSD17B13 variant also represents a potential therapeutic target, underscoring the translational relevance of genetic stratification in MASLD management. Despite these advances in understanding the genetic risk of MASLD, however, the functional mechanisms and clinical applications of these genes remain incompletely characterized and thus warrant further investigation.

### 5.2 Environmental risks as modifiable drivers of MASLD

MASLD pathogenesis is driven by a complex interplay of genetic susceptibility and environmental factors. While the genetic background remains relatively static, environmental exposures act as the predominant modifiable drivers of disease onset and progression, thereby presenting a crucial target for clinical intervention.

Energy imbalance represents the central pathophysiological mechanism underpinning MASLD, primarily comprising imbalances in nutrition and physical activity. The nutritional imbalance in MASLD is driven by a pathophysiological triad of obesity, insulin resistance (IR), and dyslipidemia, which collectively promote hepatic lipid accumulation. This process is particularly driven by visceral adiposity, a hallmark of metabolic syndrome, which in turn plays a pivotal role in the development of IR (80). IR drives the progression of MASLD by simultaneously enhancing DNL in the liver and inhibiting lipolysis in adipose tissue. This dysregulation elevates the flux of free fatty acids to the liver, leading to the accumulation of triglycerides and subsequent hepatic steatosis (81). Hepatic lipid accumulation in MASLD is fueled by two primary sources of fatty acids: systemic FFAs released from adipose tissue lipolysis and those synthesized endogenously via hepatic DNL. Importantly, heightened DNL contributes to hepatic steatosis both directly and indirectly by increasing systemic FFAs, which promotes adipose tissue expansion and further lipid overflow to the liver (82).

HTN may contribute to MASLD progression through dysregulation of the renin–angiotensin–aldosterone system (RAAS) and sympathetic nervous system (SNS) (83), which can promote insulin resistance and hepatic inflammation (80). In addition, dietary patterns and gut microbiota dysbiosis may influence MASLD development and progression by modulating hepatic lipid metabolism, insulin sensitivity, and inflammatory responses (84–86). Diets rich in saturated fats and added sugars may exacerbate metabolic dysfunction and hepatic lipid accumulation, whereas dietary modification may improve hepatic steatosis and metabolic health (87–89).

Physical inactivity, characterized by a lack of moderate-to-vigorous exercise, is a primary contributor to metabolic dysregulation. Importantly, a growing body of evidence indicates that prolonged sedentary behavior itself constitutes an independent risk factor for the development of metabolic syndrome and obesity (87). Given that a sedentary lifestyle is a significant modifiable risk factor, implementing a structured exercise regimen is a cornerstone of MASLD management. In their study, Arita et al. (90) investigated the efficacy of exercise prescriptions in MASLD/MASH patients, finding that interventions incorporating aerobic exercise were effective in reducing body weight and enhancing hepatic outcomes. Baek et al. (91) investigated the therapeutic potential of high-intensity exercise, finding it to be effective in attenuating the progression of MASLD. First-line therapy for MASLD in the United States rests on three pillars: dietary management, physical exercise, and targeted weight loss (92). This highlights the integral role of regular physical activity in controlling disease progression. Given that energy balance and body composition are central to hepatic fat accumulation, the combination of diet and exercise is paramount in MASLD management, as monotherapies often prove inadequate for addressing these core metabolic drivers. Thus, a holistic management strategy, encompassing dietary optimization and regular exercise, represents a promising approach for attenuating MASLD progression in clinical practice.

## 6 Clinical manifestations

The clinical manifestations and natural history of MASLD exhibit significant heterogeneity (Figure 4). MASLD can be broadly categorized into lean MASLD and obese MASLD. Lean MASLD has been defined as body mass index (BMI) < 23 kg/m² in the Kailuan cohort and the China Kadoorie Biobank (CKB) and <25 kg/m² in the UK Biobank (UKB) (93). Classification criteria vary slightly across regions, but the common feature of lean MASLD is its applicability to non-obese or overweight individuals.

**Figure 4:**
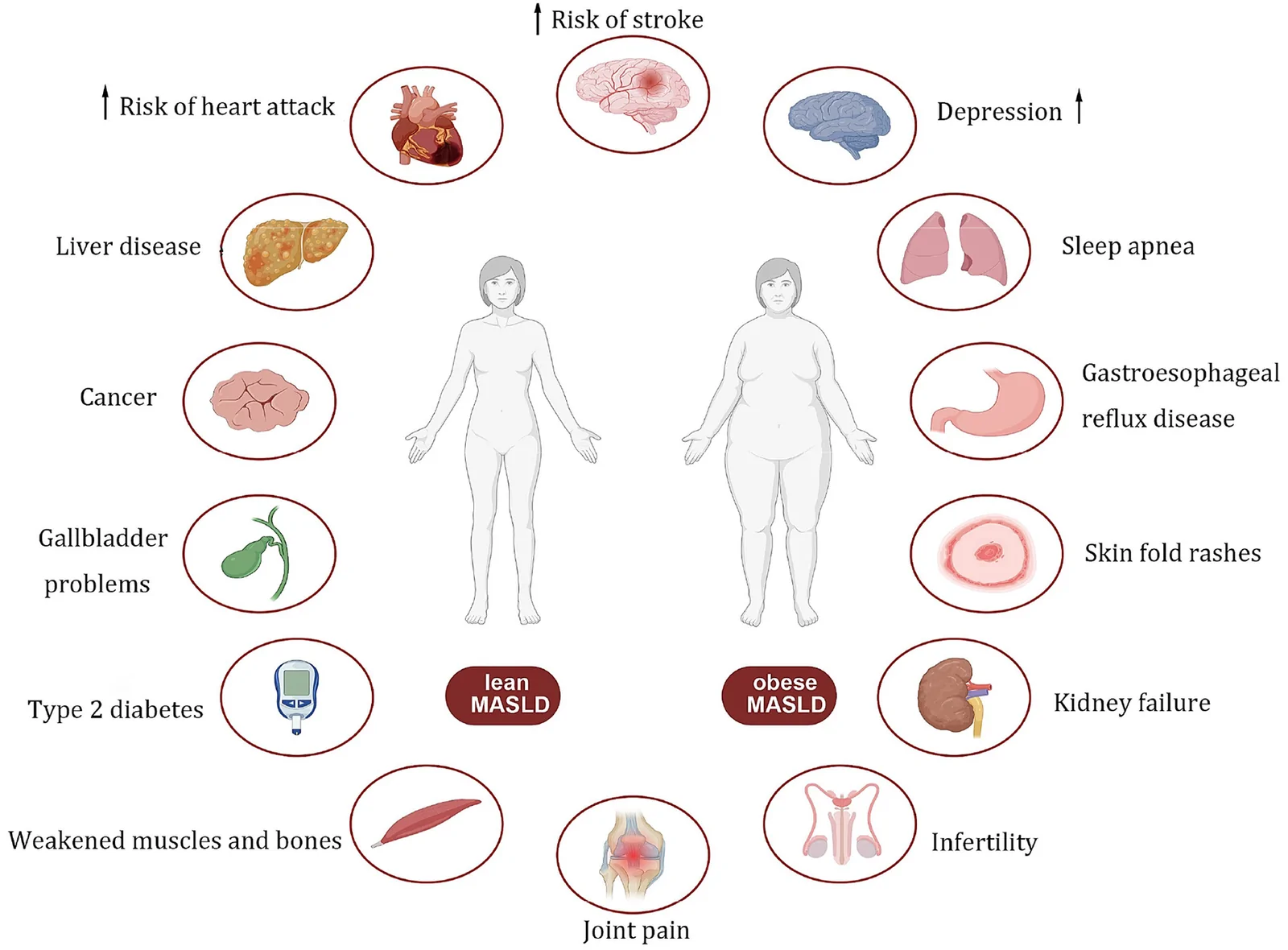
Clinical manifestations and natural course of MASLD. This illustration depicts the systemic multiorgan manifestations of MASLD stratified by body habitus, encompassing both lean and obese phenotypes. Lean MASLD is associated with an elevated risk of cardiovascular events, depression, type 2 diabetes, gallbladder disease, malignancy, and generalized muscle weakness, whereas obese MASLD additionally involves mechanical complications including gastroesophageal reflux disease, sleep apnea, skin fold rashes, renal impairment, infertility, and joint pain. Regardless of adiposity status, MASLD functions as a multisystem disorder that amplifies the risk of cardiovascular disease and hepatic carcinoma, underscoring the necessity for holistic, weight-independent surveillance strategies.

Extrahepatic complications of MASLD are highly prevalent, and virtually all organ systems may be affected. At the highest evidence level, a comprehensive meta-analysis of 8,515,431 patients by Younossi et al. (10) demonstrated that the global prevalence of MASLD was 25.24% (95% CI: 22.10-28.65%). Metabolic complications associated with MASLD include obesity (51.34%); 95% CI: 41.38–61.20%), T2DM (22.51%; 95% CI: 17.92–27.89%), arterial HTN(39.34%; 95% CI: 33.15–45.88%), hyperlipidemia (69.16%; 95% CI: 49.91–83.46%) and metabolic syndrome (42.54%; 95%CI: 30.06–56.05%). Prospective cohort studies provide robust evidence linking liver fibrosis to incident metabolic disease. Bjornstrom et al. (94), in a retrospective cohort of biopsy-proven MASLD patients without baseline diabetes, found that 31% of those with F0–F2 fibrosis and 51% of those with F3–F4 fibrosis developed diabetes during follow-up. Building on this, Ampuero et al. (95) conducted a multicenter prospective study of MASLD patients confirmed by liver biopsy and established that the severity of liver fibrosis was a driving factor for the development of metabolic outcomes such as T2DM, AHT, and dyslipidemia in metabolically healthy patients. Specifically, they observed that the risk of T2DM and AHT in patients with F3-F4 fibrosis was four times higher than in patients with F1-F2 fibrosis, with obesity conferring a further elevated risk.

Cross-sectional and population-based data offer complementary insights. In a single-center cross-sectional study of 385 MASLD participants from a tertiary center in Tehran, severe steatosis and fibrosis were prevalent, affecting 50.6% and 29.1% of the cohort, respectively (96). At the population level, given the close relationship between DM and chronic kidney disease (CKD), a Chinese population-based prospective cohort explored the relationship between MASLD and urinary albumin in patients with T2DM. Among DM patients, the prevalence of proteinuria in patients with simple hepatic steatosis (46.2%) was higher than that in patients without MASLD (41.4%), while the prevalence of proteinuria in patients with moderate to severe fibrosis (64.2%) was higher still. After adjusting for confounding factors [glycated hemoglobin (A1C), BMI and AHT], DM patients with moderate to severe liver fibrosis were found to have a higher risk of proteinuria even when the estimated glomerular filtration rate (eGFR) was ≥ 60 mL/min/1.73 m^2^ (97). In addition, long-term natural history data underscore cardiovascular disease (CVD) as the leading cause of death in MASLD. Analysis of U.S. mortality data from 2007 to 2017 revealed that CVD accounted for approximately 20% of deaths among MASLD patients, representing the most common and growing cause of mortality (98).

As a multisystem metabolic disorder with hepatic involvement, MASLD follows a dynamic and heterogeneous disease trajectory (Figure 5). The rates of disease progression and treatment response vary substantially among individuals and across different hepatic zones (20). Some patients present with simple hepatic steatosis at disease onset and maintain a relatively stable activity. However, a meta-analysis and systematic review of prospective cohorts estimated that approximately 2% of patients with simple steatosis progress to MASLD-related cirrhosis over a median follow-up of 10–15 years (99). In addition, the rate of fibrosis progression (and regression) varies considerably among patients, and fibrosis progression does not follow a linear trajectory in individual patients. Serial biopsy studies have demonstrated that while some patients experience rapid fibrosis regression following metabolic improvement, others show persistent or even worsening fibrosis despite resolution of inflammation and steatosis (100). Emerging evidence from cohort studies highlights the unpredictability of long-term outcomes. Unlike viral hepatitis, in which cirrhosis almost invariably precedes hepatocellular carcinoma (HCC), MASLD-associated HCC can arise in the absence of established cirrhosis. This observation has been corroborated by multiple retrospective and prospective cohort studies, underscoring the need for surveillance strategies that extend beyond cirrhotic patients (101).

**Figure 5:**
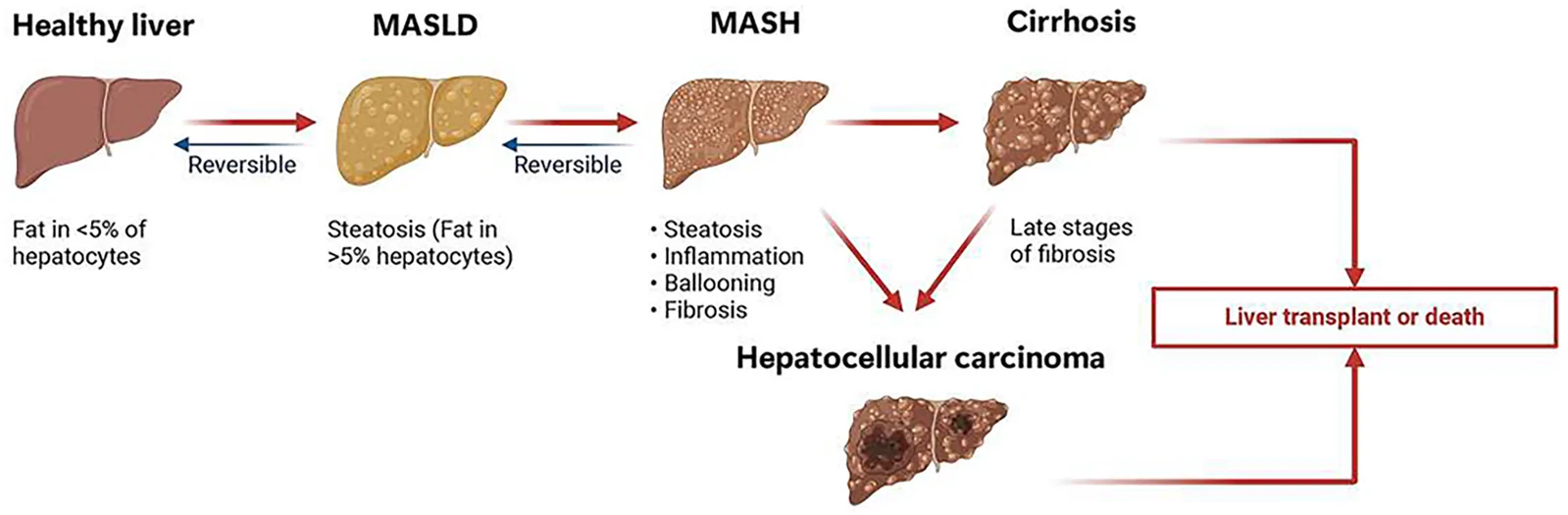
The temporal evolution of metabolic dysfunction in MASLD. This schematic illustrates the dynamic progression from a healthy liver (hepatic fat <5% of hepatocytes) through MASLD (steatosis >5%, reversible) and MASH (steatosis, inflammation, ballooning, and early fibrosis, partially reversible to cirrhosis). End-stage complications include HCC and liver failure.

## 7 Diagnostic standard

Public health responses to MASLD remain inadequate worldwide, and there is a pressing need to identify more effective diagnostic and therapeutic strategies (102). MASLD manifests as multisystem metabolic dysfunction involving the liver, and its pathogenesis, pathophysiological changes, clinical manifestations, and natural history are heterogeneous. Thus, MASLD heterogeneity necessitates distinct screening and diagnostic strategies. This review focuses on diagnostic and monitoring approaches for MASLD populations with varying risk profiles and disease stages (23) (Table 1).

**Table 1:** Detailed diagnostic testing methods of MASLD. The predictive value of this test for liver-related outcomes (such as complications of liver cirrhosis, hepatocellular carcinoma, and liver-related death) is qualitatively expressed as (+ low, ++ moderate, +++ high,? unknown). The thresholds for hepatic steatosis grading are based on the 2024 EASL–EASD–EASO Clinical Practice Guidelines for MASLD. These values represent suggested reference thresholds rather than universally validated diagnostic cut-offs.

Non-invasive Test	Biological processes reflected	Suggested thresholds		Prediction of liver related outcomes
Primary target: Hepatic steatosis				
MRI—MRI-PDFF	Lipid content	≥S1:5% ≥S2:11–18% ≥S3: 16–23%		+
VCTE: CAP	Lipid content	≥S1: 248 dB/m ≥S2: 268 dB/m ≥S3: 280 dB/m		?
US scan	Lipid content	No standardized quantitative thresholds		+
Primary target: Hepatic fibrosis		Rule-out cut-off	Rule-in cut-off	
AST/ALT ratio	Stress to hepatocytes	F3: 0.8	F3: 1.0	+
FIB-4	Stress to hepatocytes, hypersplenism	F2:0.66–0.89 F3:1.3	F2:2.67 F3:2.67	++
NFS	Stress to hepatocytes, hypersplenism, metabolic burden	F3: −1.455	F3: 0.676	++
APRI	Stress to hepatocytes, hypersplenism	F3: 0.5	F3: 1.5	++
ELF	Collagen metabolism	F3:7.7	F3:9.8	+++
VCTE: LSM	Fibrosis, extracellular volume fraction	F3:8 kPa	F3:12 kPa	+++
US—2D-SWE	Fibrosis, extracellular volume fraction	F3:8 kPa	F3: 10.5 kPa	+++
MRI—MRE	Fibrosis, extracellular volume fraction	≥F2: 3.14 kPa ≥ F3: 3.53 kPa F4: 4.45 kPa		+++
Primary target: At-risk MASH		Rule-out cut-off	Rule-in cut-off	
FAST	Stress to hepatocytes, fibrosis, lipid content	0.35	0.67	++
MAST	Stress to hepatocytes, fibrosis, lipid content	0.165	0.242	++
Corrected T1	Extracellular volume fraction	825 ms	875 ms	++

### 7.1 Diagnostic criteria for MASLD

Liver biopsy remains the gold standard for the diagnosis of MASLD. However, due to sampling bias, invasive nature and the risk of bleeding, the rate of awareness, diagnosis and treatment of MASLD are far lower than its prevalence (103). Early clinical guidelines stipulated that MASLD could be diagnosed only after excluding chronic liver disease caused by excessive alcohol consumption and other secondary causes. With further study of the pathogenesis of MASLD and its increasing prevalence, many clinical practices and studies have found that a single detection method or index is insufficient to diagnose MASLD. The exclusion-based diagnostic criteria not only lead to a considerable waste of medical resources but also delay treatment (104). In view of the complex pathophysiological characteristics of MASLD, and address the deficiencies of previous nomenclature and typing, researchers have suggested that multiple working definitions, similar to those for metabolic syndrome, should be developed to clarify the definition and diagnostic criteria of MASLD. In 2020, an international expert group put forward a new consensus statement that MASLD originates from an underlying state of metabolic dysfunction, and that MASLD should be positively diagnosed as an independent disease. The new diagnostic criteria recommend liver biopsy histology, imaging, or blood biomarker tests suggesting liver fat accumulation, combined with overweight/obesity,T2DM, and metabolic dysfunction, can be used to diagnose MASLD (20). The current diagnostic framework for MASLD, established by the 2023 international multisociety consensus and further refined in the 2024 EASL–EASD–EASO Clinical Practice Guidelines, adopts a positive diagnostic approach requiring evidence of hepatic steatosis and at least one cardiometabolic risk factor (22, 23). Hepatic steatosis can be identified through imaging or liver histology, while the specific cardiometabolic criteria are summarized in Table 2. Assessment of alcohol consumption and other potential causes of hepatic steatosis is essential for appropriate etiological classification, particularly for distinguishing MASLD from MetALD and other forms of steatotic liver disease (23).

**Table 2:** Cardiometabolic risk factors in the definition of MASLD. HbA1c, glycated hemoglobin; HDL, high-density lipoprotein; OGTT, oral glucose tolerance test.

Metabolic risk factor	Adult criteria
Overweight or Obesity	Body mass index ≥25 kg/m^2^ (≥23 kg/m^2^ in people of Asian ethnicity)
	Waist circumference ≥94 cm in men and ≥80 cm in women (Europeans) ≥90 cm in men and ≥80 cm in women (Chinese and South Asians) ≥85 cm in men and ≥90 cm in women (Japanese)
Dysglycaemia or type 2 diabetes	Prediabetes: HbA_1c_ 39–47 mmol/mol (5.7–6.4%) or fasting plasma glucose 5.6–6.9 mmol/L (100–125 mg/dL) or 2-h plasma glucose during OGTT 7.8–11 mmol/L (140–199 mg/dL) Type 2 diabetes: HbA_1c_ ≥ 48 mmol/mol (≥6.5%) or fasting plasma glucose ≥7.0 mmol/L (≥126 mg/dL) or 2-h plasma glucose during OGTT ≥11.1 mmol/L (≥200 mg/dL) *or* Treatment for type 2 diabetes
Plasma triglycerides	≥1.7 mmol/L (≥150 mg/dL) *or* lipid-lowering treatment
HDL-cholesterol	≤1.0 mmol/L (≤39 mg/dL) in men and ≤1.3 mmol/L (≤50 mg/dL) in women *or* lipid-lowering treatment
Blood pressure	≥130/85 mmHg *or* treatment for hypertension

### 7.2 Early screening of steatosis in high-risk MASLD populations

While steatosis is common in the general population, it does not confer a substantially elevated risk of clinically significant liver-related outcomes. Therefore, there is a lack of robust evidence to support population-wide screening for MASLD. Furthermore, no well-established, non-invasive detection method is currently suitable for large-scale screening purposes (23). While some analyses support the cost-effectiveness of screening for MASLD or fibrosis in the general population (105), and in high-risk groups including those with SLD (106),metabolic syndrome (107) or type 2 diabetes (108). However, a modeling study in primary care suggests that general population screening using the NAFLD fibrosis score (NFS) is not cost-effective (109). These discrepant findings underscore that the cost-effectiveness of MASLD screening is highly dependent on the target population, screening algorithm, and underlying model assumptions, highlighting the need for risk-stratified rather than universal screening strategies pending harmonized cost-effectiveness evaluations.

MASLD risk is closely linked to the burden of cardiometabolic risk factors. A rational approach to its management may therefore begin with targeted intervention and monitoring in high-risk populations. Despite significant interindividual variation, the broad natural history of MASLD is well characterized (110). MASLD generally follows a prolonged and heterogeneous course toward advanced fibrosis and cirrhosis, with the risk of liver-related complications increasing substantially at higher fibrosis stage (111, 112). Although HCC can develop in the absence of cirrhosis, the presence of cirrhosis remains the pivotal risk factor (113, 114). Therefore, preventing progression to cirrhosis represents an important therapeutic goal in MASLD, with the potential to substantially reduce the risk of subsequent liver-related complication (23, 115). In high-risk populations, such as those with cardiometabolic disease, abdominal obesity, type 2 diabetes, or abnormal liver enzymes, early screening and intervention for MASLD assume critical importance.

The critical initial step in screening for MASLD is the detection of hepatic steatosis. The two main diagnostic approaches for adults are serum-based tests and imaging, with MRI being the preferred technique. MRI provides a quantitative assessment of liver fat using the proton density fat fraction (PDFF), a capability that establishes it as the non-invasive gold standard for hepatic lipid quantification in this context (116, 117). This technique provides a valuable, non-invasive, and highly quantitative tool for the diagnosis of MASLD and the advancement of related research (118); it can accurately evaluate liver fat and demonstrates a high degree of intra-observer and inter-observer repeatability (119). Although MRI-PDFF is not equivalent to histological fatty liver grading, several studies have shown a correlation between liver steatosis measured by MRI-PDFF and fatty liver grading assessed by liver histology (120). A meta-analysis of 1,100 patients with chronic liver disease found that MRI-PDFF has an AUROC of 0.91–0.98 for histological fatty liver grades ≥1, ≥2, and ≥3 (121). Therefore, MRI-PDFF is recognized as the highest diagnostic accuracy for fatty liver in patients with MASLD and is the most accurate quantitative method for assessing liver fat content. Most clinical MRI platforms can now quantify liver lipids (122), enabling broader use, though the higher cost may limit accessibility and necessitates consideration of patients' economic circumstances.

Conventional B-mode ultrasonography is widely used to detect hepatic steatosis because of its accessibility and low cost. However, its sensitivity is limited, particularly for mild steatosis and in patients with obesity, and it does not provide reliable quantitative assessment of hepatic fat content (123). The diagnostic performance of conventional ultrasonography is also operator-dependent, further limiting its ability to detect and characterize hepatic steatosis (124). When the degree of steatosis is less than 30%, the sensitivity of ultrasound is significantly diminished (125), the sensitivity in patients with obesity is further reduced. A study of 187 patients with obesityshowed that the sensitivity of ultrasound(US) in the diagnosis of hepatic steatosis only was 49.1% (126).

In contrast, quantitative ultrasound-based methods, such as the controlled attenuation parameter (CAP) measured by vibration-controlled transient elastography (VCTE), provide a more objective assessment of hepatic steatosis (23, 123, 127). CAP has demonstrated greater sensitivity for detecting mild steatosis than conventional ultrasonography, with a reported measurement repeatability of 82% (128). Nevertheless, CAP measurements remain susceptible to technical and patient-related factors, including body habitus, diabetes, and probe selection (129). Reported measurement failure rates vary, and a study involving 5,323 patients with chronic liver disease identified female sex, a body mass index ≥30 kg/m², and metabolic syndrome as predictors of measurement failure (130). The XL probe was developed to improve the feasibility of VCTE examinations in patients with obesity (131). However, differences in CAP values between the M and XL probes may affect the selection and interpretation of diagnostic thresholds (132).

More recently, quantitative ultrasound techniques based on attenuation coefficients, including attenuation imaging (ATI), have shown promising performance in detecting and grading hepatic steatosis. A prospective multicenter study demonstrated higher diagnostic accuracy of ATI than CAP across different steatosis grades, using MRI-derived proton density fat fraction (MRI-PDFF) as the reference standard (133). Nevertheless, MRI-PDFF remains the preferred non-invasive method when accurate quantification of hepatic fat content is required (23, 123, 127). Although incidental computed tomography (CT) findings may suggest hepatic steatosis, CT is not recommended for dedicated liver fat quantification because of its limited accuracy, particularly for mild steatosis (116).

In addition to imaging techniques, biomarkers based on laboratory tests can also predict the degree of liver steatosis and serve as an auxiliary reference for diagnosing hepatic steatosis. Hepatic steatosis is a typical histological change in the early stage of MASLD. The Steato Test diagnostic model incorporates parameters such as BMI, serum total bilirubin, GGT, *α*2-macroglobulin (*α*2 m), transferrin, ALT, and apolipoprotein AI (Apo AI), as well as total cholesterol, TG, and glucose levels adjusted for age and sex. When liver biopsy was used as the reference standard, these parameters demonstrated moderate accuracy in diagnosing hepatic steatosis (AUROC: 0.79–0.80) (134). The MASLD liver fat score (Massachusetts General Hospital Liver Fat Score, MGH LFS) was based on MRI-PDFF as the reference standard. The model incorporated metabolic syndrome and T2DM status, serum fasting insulin, and AST levels, as well the AST/ALT ratio. The score demonstrated good accuracy in the diagnosis of MASLD (defined as liver fat content≥5.56%) (AUROC: 0.86–0.87) (135). However, serum insulin levels and non-routine laboratory tests included in the formula may limit its widespread clinical application. Moreover, the inclusion of collinear indicators (AST level and AST/ALT ratio) in the formula may lead to overfitting of the results. The fatty liver index (FLI) incorporates BMI, waist circumference, serum TG, and gamma-glutamyl transferase (GGT) levels (136), and is also associated with insulin resistance. With conventional ultrasound as the reference standard, FLI demonstrated moderate accuracy in the diagnosis of MASLD (AUROC: 0.84) and was able to predict all-cause, liver-related tumor and overall mortality (137). In a large cohort of 10,000 healthy subjects, researchers proposed a hepatic steatosis index (HSI) incorporating the serum aspartate aminotransferase (AST) to alanine aminotransferase (ALT) ratio, BMI, sex, and diabetes status. Similarly, when traditional ultrasound was used as the reference standard, HSI demonstrated moderate accuracy in the diagnosis of MASLD (AUROC: 0.81) (138). Since the reference standard for FLI and HSI is conventional US rather than a more sensitive modality, the results may be affected by low sensitivity and repeatability (139). Overall, Steato Test performed better in terms of accuracy and repeatability, while MGH LFS, FLI, and HSI offered certain advantages in MASLD screening. However, these methods cannot directly assess the degree of liver fibrosis and inflammation.

### 7.3 Diagnostic strategies for liver fibrosis of MASLD

Beyond the initial detection of hepatic steatosis, assessment of liver fibrosis is a critical component in the management of patients diagnosed with MASLD. This evaluation is essential because the stage of fibrosis is strongly associated with liver-related outcomes (140) and serves as a key predictor of disease progression, thereby directly informing clinical decision-making and therapeutic strategy. In adults diagnosed with MASLD, a sequential, stepwise approach is recommended for fibrosis assessment. Initial evaluation should incorporate the validated non-invasive scoring system, the Fibrosis-4 (FIB-4) index. Subsequently, non-invasive imaging modalities, such as liver stiffness measurement via elastography, are advised to further stratify fibrosis stage. In cases of persistent diagnostic uncertainty or in high-risk individuals, serum biomarkers reflective of collagen turnover may be considered as an alternative to imaging for identifying advanced fibrosis (23).

The FIB-4 index is the most extensively utilized non-invasive tool for fibrosis assessment. Its diagnostic utility, however, is constrained within an indeterminate range (typically 1.3–2.67), and its accuracy is reduced in specific populations, including the elderly and patients with type 2 diabetes (141). Consequently, as a standalone test, FIB-4 can yield a high rate of false-positive results, particularly in populations with a low disease prevalence. Notably, a lower threshold of approximately 2.0 should be applied for individuals over 65 years of age. Both FIB-4 and the NFS demonstrate moderate accuracy for predicting advanced fibrosis (stage ≥ F3), with reported areas under the receiver operating characteristic curve of approximately 0.77 and 0.75, respectively (142). Both FIB-4 and NFS demonstrate suboptimal performance in individuals under 35 years of age (143). In recent years, machine learning and artificial intelligence have been applied to develop optimized scores from multivariate parameter inputs. While derivative scores such as FIB-6 may not be defined by a straightforward mathematical formula, they hold promise for improved diagnostic accuracy (144). Several novel scoring systems, including the liver risk score, SAFE score, and MAF-5 score, are currently under development for application in population-based settings (145–147). Future studies are warranted to further evaluate their performance with respect to diagnostic accuracy, reproducibility, and cost-effectiveness. The sequential diagnostic algorithm (FIB-4 > elastography > ELF/biopsy) exemplifies how heterogeneity in fibrosis risk drives tiered diagnostic intensity. This risk-adapted approach minimizes unnecessary invasive procedures in low-risk patients while ensuring timely specialist referral for high-risk individuals, aligning with precision medicine principles.

Liver fibrosis alters the mechanical stiffness of liver tissue, which can be quantitatively assessed using imaging-based elastography techniques, such as ultrasound-based or magnetic resonance elastography (148). Specialized ultrasound equipment enables transient elastography. Through vibration-controlled transient elastography (VCTE), liver stiffness measurement (LSM) and CAP values can be obtained, providing reliable estimates of fibrosis severity and steatosis grade (149, 150). Additional ultrasound-based elastography techniques available on standard ultrasound systems encompass two-dimensional shear wave elastography (2D-SWE) and point shear wave elastography (pSWE) (151). These techniques exhibit diagnostic performance comparable to VCTE in staging liver fibrosis (152, 153). However, their applicability is often limited in adults with class 2 obesity, where reliable examination may not be feasible (154).

The measurement of serum biomarkers related to extracellular matrix turnover, such as collagen-derived components, can serve as valuable indicators of liver fibrosis. The Enhanced Liver Fibrosis (ELF) test, for instance, quantifies three specific serum markers of extracellular matrix metabolism to yield a composite score for fibrosis assessment (155). A meta-analysis incorporating 63 studies demonstrated that the EL*F* test has good diagnostic accuracy for detecting significant fibrosis, advanced fibrosis, and cirrhosis, with area under the receiver operating characteristic curve values of 0.811, 0.812, and 0.810, respectively (156). A cohort study involving 3,378 patients indicated that the EL*F* test, used alone or in combination with FIB-4, facilitates liver fibrosis screening in both the general population and high-risk groups. Compared with FIB-4 and NFS, it reduces unnecessary referrals without compromising the detection of true positive cases (157).

### 7.4 Diagnostic strategies for MASH

At present, liver biopsy remains the gold standard for the diagnosis of MASLD, and it is also the only method that can reliably distinguish between simple steatosis and MASH, as well as identify the cause of liver injury (158). Although non-invasive methods provide valuable information regarding liver status (141), they currently lack the capability to accurately evaluate the histopathological features of MASH, such as hepatocyte ballooning or lobular inflammation. Histological assessment remains central to the phenotypic classification of MASH in clinical research (23).

Currently, the most commonly used grading and staging system for MASH is the Brunt system. This system combines histological features such as steatosis, ballooning degeneration, and inflammation to grade the disease, as mild moderate, or severe, and uses the pattern and location of fibrosis to classify the fibrosis stage into four stages (159). The NAS (NAFLD Activity Score) is activity score: defined as steatosis (0–3), lobular inflammation (0–3) and ballooning (0–2). The individual scores are summed to obtain a total score (range 0–8). The NAS is helpful for assessing the severity of MASLD but cannot be used to diagnose MASH (160). In addition, Bedossa et al. (161) proposed a third scoring system similar to the MASH CRN, in which the researchers introduced an algorithm to classify MASLD diagnoses as either MASLD with simple steatosis or MASH. The algorithm proposes that MASLD with simple steatosis or MASH can be diagnosed only when the steatosis score is at least 1. The distinction between MASLD with simple steatosis and MASH depends on the lobular inflammation and ballooning scores.

Furthermore, some scoring systems based on blood biomarkers may be helpful in identifying MASH patients at risk of disease progression. MASH is driven hepatocyte injury resulting from apoptosis, inflammation, oxidative stress, and aberrant adipokine signaling as steatosis progresses, involving multiple hormonal, cellular and molecular perturbations. This heterogeneity and complexity render MASH a condition with numerous potential biomarkers (162). At present, ALT remains the most widely used blood marker for the clinical evaluation of chronic liver diseases, including MASH. However, ALT alone has poor predictive value for MASH. Studies have shown that MASH correlates best with liver TG levels (163). The MASH Test included MASLD patients who underwent liver biopsy and constructed a model based on age, sex, height, weight, serum TG, cholesterol, ApoAI, haptoglobin, GGT, ALT, AST, and total bilirubin. This model demonstrated moderate accuracy for the diagnosis of MASH (AUROC: 0.69–0.79) (164). Compared with simple steatosis, hepatocyte death caused by apoptosis or necroptosis is increased in MASH patients, leading to the release of cytokeratin 18 (CK18) fragments. CK18, combined with the serum apoptosis-mediated surface antigen FAS, and demonstrated good accuracy in the diagnosis of MASH (AUROC: 0.79–0.93) (165).The MASH Diagnostics model constructed by Younossi et al. (166), incorporating diabetes, sex, BMI, serum TG, total CK18, and CK18 M65 fragments, demonstrated good accuracy (AUROC: 0.81). Shen et al. (167) adopted a strategy of sequentially incorporating CK18 and fibroblast growth factor 21 (FGF21), achieving high specificity and sensitivity > 90% for the diagnosis of MASH, albeit at the cost of approximately 70% of patients remaining in an indeterminate category. Moreover, the sample sizes of the above original studies are small, and the subjects are predominantly patients with obesity. Therefore, the diagnostic efficiency of these indicators lacks independent verification, and further research in large sample sizes, across different ethnic groups and in non-obese populations is needed. Secondly, the optimal diagnostic cut-off value of each maker remains uncertain, and there is a lack of relevant research on the influencing factors and comparability between different monitoring methods. Furthermore, no studies have evaluated the dynamic properties of these biomarkers, and whether they can be used to monitor disease progression or evaluate therapeutic response in MASH remains inconclusive. Further research on MASH-related markers should demonstrate cost-effectiveness and direct impact on patient management before widespread adoption in clinical practice.

## 8 Therapeutic strategies of MASLD

The management of MASLD encompasses three key approaches: lifestyle modification, pharmacological therapy, and surgical intervention (Figure 6).

**Figure 6:**
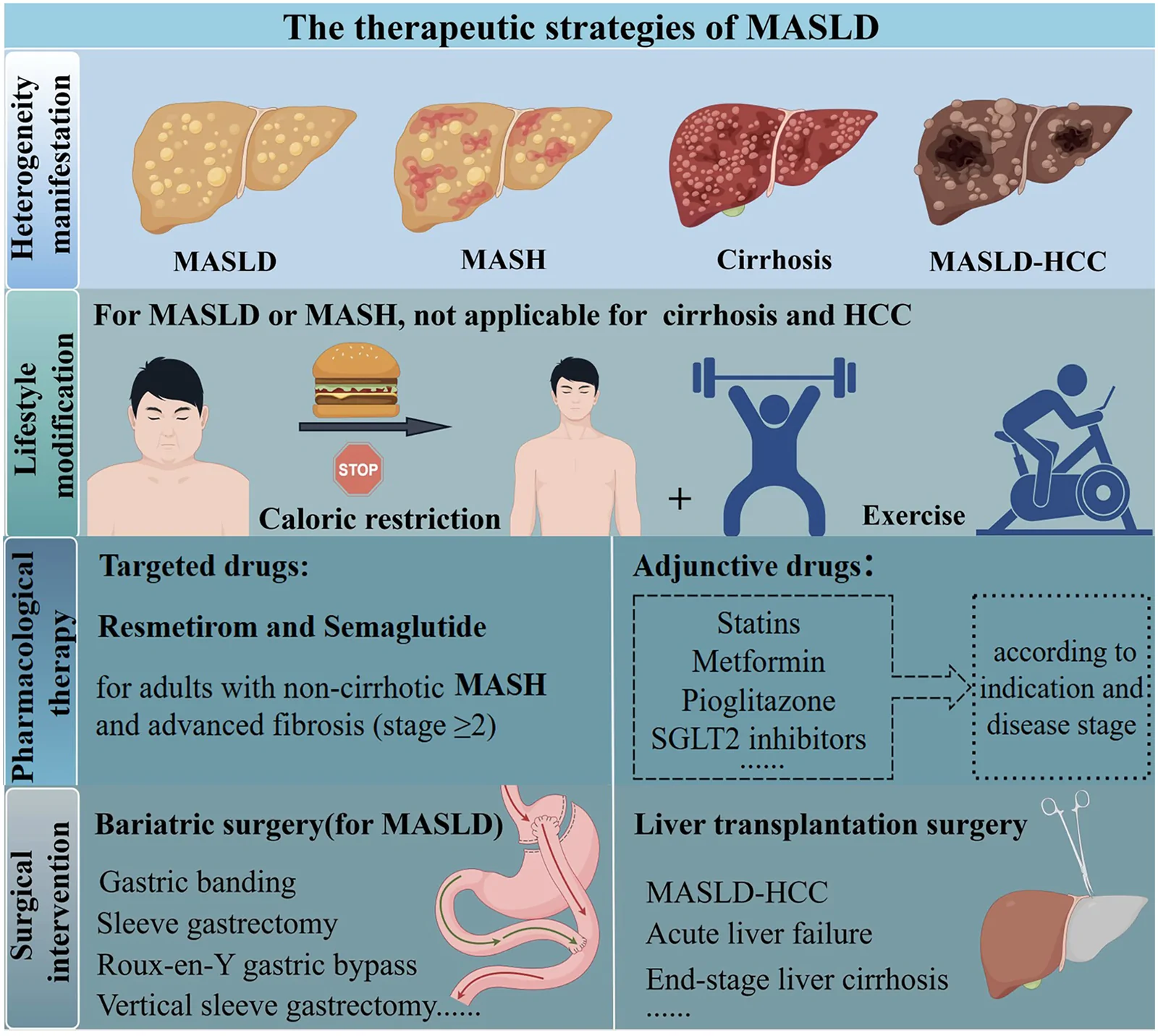
This figure summarizes the principal therapeutic approaches for MASLD, MASH, cirrhosis, and MASLD-related hepatocellular carcinoma (HCC), including lifestyle modification, pharmacological treatment, and surgical interventions. Lifestyle modification, particularly dietary intervention and physical activity, remains a cornerstone of MASLD management, with recommendations adapted to disease severity. MASH-targeted pharmacotherapy includes resmetirom and semaglutide, where locally approved, for adults with noncirrhotic MASH and moderate-to-advanced fibrosis (F2–F3). Adjunctive pharmacotherapy, including statins, metformin, pioglitazone, and sodium–glucose cotransporter-2 (SGLT2) inhibitors, is primarily directed toward the management of cardiometabolic comorbidities and should be selected according to clinical indications and disease stage. Surgical interventions include bariatric surgery for eligible patients with MASLD and liver transplantation for selected patients with decompensated cirrhosis or HCC.

### 8.1 Lifestyle modification

Current guidelines recommend that adults with MASLD adopt dietary and behavioral interventions to achieve weight loss, thereby improving liver-related outcomes (23). Multiple randomized controlled trials (RCTs) and meta-analyses have consistently demonstrated that weight loss achieved through caloric restriction improves key biomarkers of MASLD, including liver enzyme levels, hepatic steatosis, steatohepatitis, and fibrosis, independently of increases in physical activity (168–170). The extent of improvement in liver injury biomarkers exhibits a dose-dependent relationship with the magnitude of weight loss (170). Nevertheless, evidence from these same trials regarding the impact of lifestyle-mediated weight loss on advanced fibrosis or cirrhosis remains limited, owing to the relatively small number of patients with advanced fibrosis enrolled in most trials and a general lack of prespecified subgroup analyses (171). Therefore, weight-loss interventions are not currently recommended for patients with advanced fibrosis or cirrhosis.

Several dietary approaches have demonstrated benefits in promoting weight reduction and improving MASLD. Notably, RCTs and systematic reviews have shown that low-calorie, low-carbohydrate, and low-fat diets can each effectively reduce hepatic lipid content and associated biomarkers (172, 173). In addition, a systematic review and meta-analysis further indicated that coffee consumption is associated with a dose-dependent improvement in liver enzyme levels, including among individuals at elevated risk for liver disease (174). Meanwhile, another meta-analysis found that consuming more than 3 cups of coffee per day, but no fewer than 2 cups, offered significant protection (175). Other specific dietary regimens have also been shown to confer modest benefits in alleviating MASLD. Evidence from prospective cohort studies and RCTs suggests that the Mediterranean diet offers added benefits for both hepatic steatosis and cardiometabolic risk profiles, with superior long-term sustainability (84, 176). The ketogenic diet achieves a greater reduction in hepatic triglycerides by inducing a fasting-like metabolic state (177). However, limited trial data preclude conclusions regarding the effectiveness and safety of the extremely low-carbohydrate ketogenic diet (178). Time-restricted eating, an intermittent fasting protocol in which food consumption is limited to a prescribed period each day, has been examined in several pilot RCTs (179). In a 16-week randomized controlled trial, time-restricted feeding achieved a 25.8% reduction in hepatic fat in MASLD, with effects on weight loss, body composition, and metabolic markers similar to those of calorie restriction (179).

Sedentary behavior is an independent risk factor for MASLD (180) and is further linked to an increased likelihood of disease progression (181). Multiple randomized controlled trials and subsequent meta-analyses have established that exercise can ameliorate hepatic steatosis in MASLD without requiring dietary modification or significant weight reduction (182). A randomized controlled trial showed that a 3-month aerobic exercise program (three 90-minute sessions per week) reduced hepatic steatosis and fibrosis, an effect independent of weight loss (183). Multiple randomized controlled trials have investigated the efficacy of various exercise protocols, including aerobic, resistance, and high-intensity interval training in MASLD. A meta-analysis of available evidence indicated that physical activity did not significantly improve outcomes in children and adolescents with MASLD (184). In contrast to pharmacotherapies that effectively reduce liver fat, a standardized, optimal exercise prescription for patients with fibrosis has yet to be established (185, 186). A meta-analysis of 19 RCTs comparing high-intensity interval training (HIIT) to moderate-intensity continuous training (MICT) found both aerobic exercise modalities to be equally effective in reducing hepatic fat (187). Given the limited trial data, the effect of exercise training on liver histology, independent of other interventions, remains unclear (188, 189). However, randomized controlled trials conducted in patients with MASLD have shown that exercise can improve quality of life, cardiovascular health, reduce visceral fat, and enhance muscle mass and function, even in advanced liver disease (190, 191). Furthermore, increased physical activity is associated with reduced risks of liver fibrosis (as assessed by non-invasive markers), cirrhosis, hepatocellular carcinoma, and all-cause mortality, as shown in numerous observational studies (192–195). Collectively, the benefits of increased physical activity and reduced sedentary behavior for hepatic and cardiometabolic outcomes, which are independent of weight loss, argue for the routine promotion of these behaviors in standard patient care (181, 196).

### 8.2 Pharmacological therapy

Resmetirom and semaglutide are currently the only therapies approved by the U.S. Food and Drug Administration (FDA) for use in non-cirrhotic patients with MASH and advanced fibrosis. Resmetirom is a thyroid hormone receptor-*β* (THR-*β*)-selective agonist with oral bioavailability and hepatic targeting, designed for the treatment of MASH (197). In light of the evolving treatment landscape for MASH and supported by phase 3 trial data demonstrating histological improvement and acceptable safety, resmetirom represents a targeted treatment option. Therefore, upon local regulatory approval, its use should be considered for adults with non-cirrhotic MASH and advanced fibrosis (stage ≥2), consistent with the approved label (23). In the phase 3 MAESTRO-NASH trial, 966 patients with obesitywith biopsy-confirmed MASH and fibrosis stages F1–F3 were randomized to receive resmetirom (80 mg or 100 mg daily) or placebo for 52 weeks. Resmetirom at 80 mg and 100 mg achieved histological resolution of steatohepatitis without worsening of fibrosis in 25.9% and 29.9% of patients, respectively, compared with 9.7% in the placebo group (both P < 0.001). Fibrosis improvement by ≥1 stage without worsening of steatohepatitis occurred in 25.9% of the 100 mg group vs. 14.2% of the placebo group (P < 0.001); similar improvement was observed in the 80 mg group (198). Prespecified secondary analyses demonstrated that the efficacy of resmetirom was independent of concomitant use of sodium-glucose cotransporter 2 inhibitors (SGLT2) or glucagon-like peptide-1 receptor agonists (GLP1-RA). Moreover, patients who achieved ≥5% weight loss derived greater benefit, with higher rates of MASH resolution (56.6% compared with 33.8%), fibrosis improvement (40.6% compared with 31.5%), greater relative reduction in MRI-PDFF (-69% compared with −46%), and greater reduction in liver stiffness (−4.6 kPa compared with −2.3 kPa) (199).

In addition to resmetirom, the FDA conditionally approved semaglutide (2.4 mg/week) for the treatment of MASH with moderate to advanced fibrosis. In the phase 3 ESSENCE trial, 534 patients received semaglutide and 266 received placebo. Semaglutide led to MASH resolution without worsening of fibrosis in 62.9% of patients, compared with 34.3% in the placebo group (P < 0.001). Fibrosis improvement by ≥1 stage without worsening of steatohepatitis occurred in 36.8% of the semaglutide group vs. 22.4% of the placebo group (P < 0.001). Bodily pain scores did not differ significantly between groups. However, gastrointestinal adverse events were more frequent with semaglutide. For patients with cirrhosis due to MASH, no targeted drug therapies are currently recommended (200).

Beyond approved MASH-directed therapies, several agents have been investigated for their potential histological benefits. Vitamin E has demonstrated improvements in steatohepatitis in selected patients without diabetes, as shown in the PIVENS trial, but a consistent antifibrotic benefit has not been established. Epidemiological studies suggest that higher dietary vitamin E intake may help prevent the onset of MASLD, as defined by standard clinical and radiographic criteria, especially among those with type 2 diabetes (201). Studies of vitamin E supplements have yielded inconclusive results concerning cardiovascular mortality and prostate cancer risk, and have not shown benefit in intervention studies (202–204). 24-Norursodeoxycholic acid (norursodeoxycholic acid), a novel synthetic derivative of UDCA, has emerged as a compound with choleretic, anti-inflammatory, and anti-fibrotic activities in preclinical testing (205). The MASH trial is currently ongoing, with preliminary results demonstrating improvements in key endpoints including ALT levels and hepatic steatosis (206). Pegozafermin, a long-acting fibroblast growth factor 21 (FGF21) analogue, has also shown promising results in patients with noncirrhotic MASH and stage F2–F3 fibrosis. In the phase IIb ENLIVEN trial, fibrosis improvement of at least one stage without worsening of steatohepatitis was achieved in 26% and 27% of patients receiving pegozafermin at doses of 30 mg and 44 mg, respectively, compared with 7% in the placebo group (207) (Figure 7).

**Figure 7:**
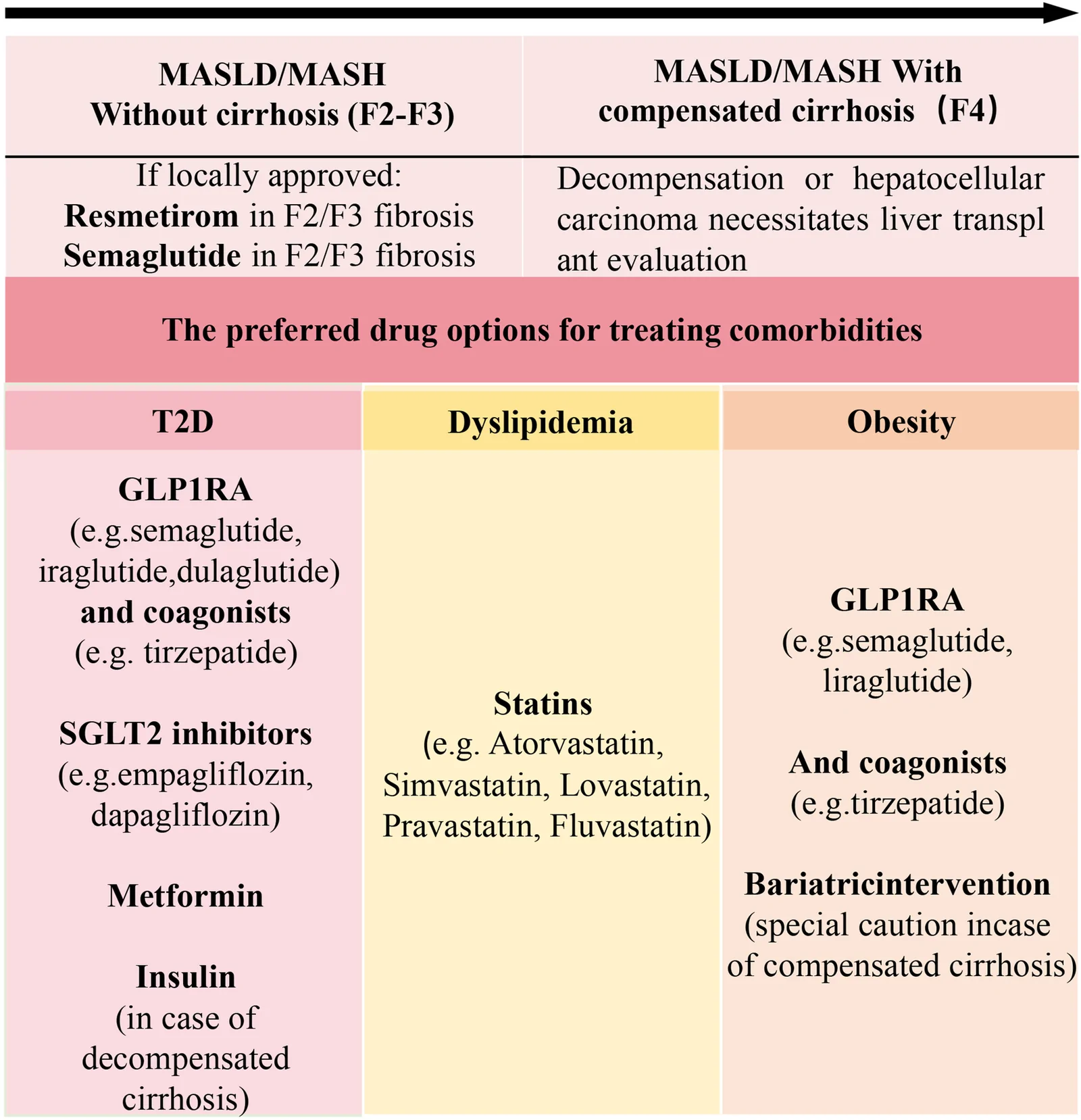
Stratified medication strategies for MASLD. This table outlines the stage-specific therapeutic approach for MASLD, differentiating between non-cirrhotic (F2–F3) and compensated cirrhosis (F4) stages. In F2–F3 fibrosis, resmetirom and semaglutide are recommended if locally approved, progression to F4 necessitates evaluation for liver transplantation upon decompensation or HCC development. Glucagon-like peptide-1 receptor agonists (GLP1RA) and sodium-glucose cotransporter-2 inhibitors (SGLT2) are indicated for type 2 diabetes (T2D), statins for dyslipidemia, and GLP1RA or bariatric interventions for obesity, with special caution advised for the latter in compensated cirrhosis.

### 8.3 Surgical interventions

Bariatric surgery is recommended for adults with non-cirrhotic MASLD who meet the criteria, as it is associated with durable liver disease benefits, alleviation of type 2 diabetes, and improved cardiometabolic profiles. The most common weight loss surgeries include restrictive procedures such as gastric banding and sleeve gastrectomy, or techniques that divert the distal part of the stomach contents to the small intestine (gastroenterostomy), such as Roux-en-Y gastric bypass, biliopancreatic diversion, or single anastomosis gastric bypass. Prospective cohort studies have demonstrated that bariatric surgery results in sustained weight reduction, remission of type 2 diabetes, improvement of cardiovascular risk factors, and a lower risk of cancer (including liver cancer) (208–210). A meta-analysis of 30 studies involving 3,134 patients reported that bariatric surgery reduced BMI to a mean ratio of 0.79 of baseline values, while MRI-PDFF demonstrated a 72% reduction in intrahepatic fat (mean ratio 0.28) (211).

Importantly, the magnitude of histological improvement varies across surgical procedures and outcome measures. In a prospective study involving 40 patients with paired liver biopsies, RYGB produced a greater reduction in histological steatosis grade than SG at 12 months (mean change = −0.91 and −0.33; *P* = 0.007), whereas both procedures significantly improved the NAFLD Activity Score without a statistically significant reduction in fibrosis stage (212). In the multicenter randomized BRAVES trial, steatohepatitis resolution without worsening of fibrosis was achieved at 1 year in 56% of patients undergoing RYGB and 57% undergoing SG, compared with 16% receiving lifestyle modification and best medical care. Fibrosis improvement of at least one stage without worsening of steatohepatitis occurred in 37%, 39%, and 23% of patients, respectively (213). A recent meta-analysis of three randomized trials involving 322 patients found that both RYGB and SG improved fibrosis-related outcomes, without a statistically significant difference between the procedures (OR for RYGB vs. SG: 1.63; 95% CI: 0.94–2.81) (214). Longer-term evidence also suggests sustained histological benefits: in a prospective cohort, steatohepatitis resolved in 84% of patients and fibrosis disappeared in 56% of patients with follow-up biopsies 5 years after bariatric surgery (215).

Moreover, multiple observational studies have established that significant weight reduction is associated with improved liver enzyme levels and related biochemical parameters (216–220). Two meta-analyses incorporated 30 studies, including data from over 3,700 patients with MASLD/MASH who underwent bariatric surgery. The results indicated that Roux-en-Y gastric bypass was most strongly associated with improved steatohepatitis and showed superior efficacy for histological outcomes compared with other bariatric procedures (221, 222). Roux-en-Y gastric bypass can lead to histological improvement in liver fibrosis in approximately 30% of patients. Rates of improvement in both steatosis and fibrosis appear to be even higher among Asian populations (222).

Together, these findings underscore the need to individualize MASLD management according to disease etiology, fibrosis stage, metabolic phenotype, and coexisting conditions. Building on these considerations, Figure 8 presents a conceptual clinical decision framework that integrates etiological assessment, fibrosis risk stratification, and heterogeneity profiling to inform stage-appropriate management.

**Figure 8:**
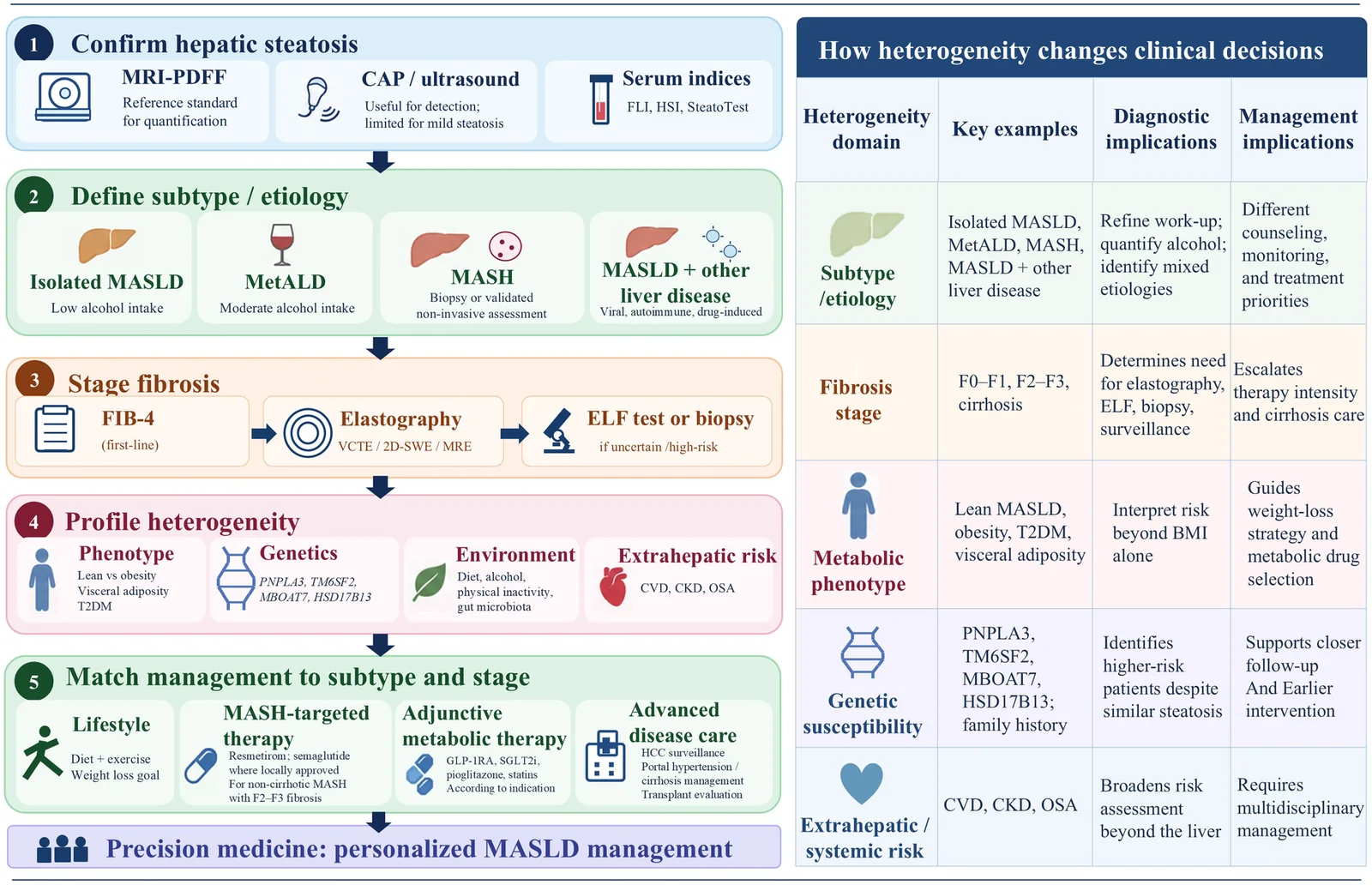
Heterogeneity-informed clinical decision framework for MASLD. The left panel illustrates a five-step framework integrating the identification of hepatic steatosis, etiological classification, fibrosis staging, heterogeneity profiling, and individualized management. Clinical assessment incorporates metabolic phenotypes, genetic susceptibility, environmental exposures, and extrahepatic comorbidities to inform risk stratification and treatment selection. Management strategies include lifestyle modification, MASH-targeted pharmacotherapy for eligible patients with noncirrhotic disease, treatment of cardiometabolic comorbidities, and management of advanced liver disease. The right panel summarizes how distinct dimensions of MASLD heterogeneity influence diagnostic priorities and therapeutic decisions. Together, these components provide a structured approach to integrating disease heterogeneity into clinical management.

## 9 Discussion

In conclusion, MASLD epitomizes the challenge of translating a heterogeneous, multisystem metabolic disorder into precision clinical care. This review has traced the field's evolution from the exclusion-based NAFLD/NASH paradigm to the positive-diagnosis MASLD framework with four principal subtypes: isolated MASLD, MetALD, MASH, and MASLD with other liver diseases (Figure 1) and further dissected the drivers of heterogeneity across three interlocking layers: (i) hepatic lobular spatial biology and lipid-overload/inflammatory cascades; (ii) a multilayered interplay of genetic (PNPLA3, TM6SF2, MBOAT7, GCKR, HSD17B13, CNV), epigenetic, and environmental modifiers, conceptualized here as an iceberg model (Figure 3); and (iii) divergent clinical trajectories, from lean vs. obese phenotypes to extrahepatic manifestations (CVD, T2DM, CKD) and variable natural histories (Figures 4, 5). Diagnostic and therapeutic strategies must accordingly move beyond a “one-size-fits-all” approach: non-invasive steatosis/fibrosis pathways (MRI-PDFF, CAP, FIB-4, elastography) and biomarker panels enable risk-adapted screening, while emerging targeted therapies, resmetirom, semaglutide, and pegzafermin, alongside lifestyle and bariatric approaches, can be matched to disease stage and subtype (Figures 6, 7).

All in all, MASLD is a heterogeneous disorder in which disease progression and clinical outcomes are influenced by the interplay of genetic susceptibility, metabolic dysfunction, environmental exposures, and coexisting liver diseases. Importantly, these differences have implications for clinical management beyond conventional disease classification. As illustrated in Figure 8, a heterogeneity-informed approach should integrate etiological assessment, fibrosis staging, metabolic phenotyping, and extrahepatic risk evaluation to guide individualized management.

Etiological heterogeneity influences the diagnostic evaluation and subsequent management of steatotic liver disease. Although hepatic steatosis and at least one cardiometabolic risk factor establish the diagnostic basis for MASLD, alcohol consumption and potential coexisting liver diseases require further assessment to identify overlapping etiologies. Metabolic phenotypes also influence clinical evaluation. For example, individuals with lean MASLD may exhibit visceral adiposity and insulin resistance despite having a normal body mass index, highlighting the need to assess metabolic dysfunction rather than relying on body weight alone.

Fibrosis severity remains a major determinant of liver-related prognosis and treatment selection. Sequential non-invasive assessment using FIB-4 and elastography can identify patients requiring further evaluation, while evidence of significant fibrosis may influence eligibility for MASH-targeted therapy. In adults with noncirrhotic MASH and F2–F3 fibrosis, resmetirom and semaglutide may be considered where locally approved and clinically indicated. In contrast, patients with cirrhosis require management focused on portal hypertension, hepatocellular carcinoma surveillance, and prevention of hepatic decompensation. Concurrently, cardiometabolic comorbidities, including T2DM, cardiovascular disease, and chronic kidney disease, should inform the selection of adjunctive therapies and multidisciplinary care.

Genetic and environmental factors may further refine the interpretation of disease heterogeneity. Variants in *PNPLA3*, *TM6SF2*, *MBOAT7*, and *HSD17B13* are associated with differences in susceptibility to liver injury and disease progression, whereas dietary patterns, alcohol exposure, and gut microbiota alterations may modify metabolic and inflammatory responses. Integrating these factors with clinical phenotypes may improve the identification of patients at increased risk despite similar degrees of hepatic steatosis. However, genotype-informed treatment selection remains an emerging approach rather than an established component of routine MASLD management.

Taken together, these considerations form the basis of the proposed heterogeneity-informed clinical framework (Figure 8), which links disease etiology, fibrosis severity, metabolic phenotype, and genetic and environmental modifiers to diagnostic priorities and management strategies. Rather than treating MASLD as a uniform clinical entity, this framework emphasizes the distinct contributions of disease stage and patient-specific risk factors to clinical decision-making.

A key distinction of this review is its focus on translating heterogeneity characterization into actionable clinical decisions. While the NEJM review by Targher et al. (1) provides an authoritative disease overview and the JAMA review by Tilg et al. (223) summarizes current evidence, neither systematically addresses how the four MASLD subtypes differ in their diagnostic pathways and therapeutic responses. The stratification framework proposed herein (Figure 8) bridges this gap by providing a clinical decision tool that integrates subtype classification, fibrosis staging, genetic risk assessment, and treatment matching, a dimension that complements rather than duplicates existing reviews.

However, several limitations of this narrative review should be acknowledged. First, as a non-systematic narrative synthesis, the literature search, though targeted and reproducible, may not have captured all relevant studies, introducing potential selection bias. Second, the included studies exhibit considerable heterogeneity in study design, sample size, patient demographics, and outcome definitions, which precludes quantitative pooling of results. Third, the absence of a meta-analysis limits our ability to provide effect estimates or assess publication bias, reducing the precision of any implied causal relationships. Despite these constraints, the clinical stratification framework proposed herein, grounded in etiology, pathogenesis, risk factors, diagnostic criteria, clinical manifestations, and therapeutic options, offers a pragmatic and actionable roadmap for clinicians. Future prospective studies and systematic reviews with meta-analyses are warranted to validate and refine this approach, moving toward truly personalized management of MASLD.

## 10 Future directions and research gaps

Future research should establish whether incorporating genetic, environmental, and metabolic heterogeneity into existing clinical models improves risk prediction and treatment selection in MASLD. Large, ancestrally diverse prospective cohorts are needed to assess the additional prognostic value of genetic risk scores and gene–environment interactions beyond established fibrosis-based assessments (23, 45). Longitudinal studies should also clarify disease trajectories in lean MASLD, MetALD, and patients with overlapping liver disease etiologies to inform subgroup-specific risk assessment. Emerging non-invasive biomarkers and multimodal artificial intelligence models require prospective evaluation of their ability to predict hepatic decompensation, hepatocellular carcinoma, and liver-related mortality beyond existing clinical tools (23, 224). Randomized trials incorporating prespecified genetic and metabolic subgroup analyses are also needed to identify predictors of treatment response and determine whether heterogeneity-informed therapeutic strategies improve clinical outcomes (23, 45).

## Supplementary material

SUPPLEMENTARY FIGURE S1 Evidence landscape of 224 cited references. (A) Evidence pyramid depicting the hierarchical distribution of cited literature from Level I (systematic reviews/meta-analyses, highest quality) to Level V (expert consensus, lowest quality). (B) Quantitative breakdown showing proportional distribution and detailed counts. High-level evidence (Levels I-II) accounts for 21.4% (48/224) of citations; observational studies (Level III) constitute the largest category at 50.9% (114/224), reflecting the broad empirical foundation of this review.

SUPPLEMENTARY TABLE S1 Evidence quality assessment of core references informing the MASLD stratification framework (n = 30). References were selected to represent the highest level of evidence for each pillar of the review. Evidence level follows the Oxford Centre for Evidence-Based Medicine 2011: 1 = systematic review of RCTs; 2 = individual RCT or SR of observational studies; 3 = cohort or case-control; 4 = cross-sectional or mechanistic; 5 = expert consensus/guideline. This assessment supports the narrative synthesis but does not replace a formal GRADE evaluation.

SUPPLEMENTARY TABLE S2 List of abbreviations. This table lists the full definitions of key acronyms and technical terms referenced throughout the manuscript, encompassing clinical diagnostic tools, disease nomenclature, genetic and molecular markers, and pharmacological agents, to facilitate reader comprehension.
