# Supplementary material for: Navigating MASLD heterogeneity: from metabolic risk to precision stratification
Source: Front Med (Lausanne). 2026 Oct 30;13:1966460. doi: 10.3389/fmed.2026.1966460 (PMC13760200; doi:10.3389/fmed.2026.1966460)
Supplement: Supplementary file 1 [file Image1.pdf]

A

Evidence hierarchy

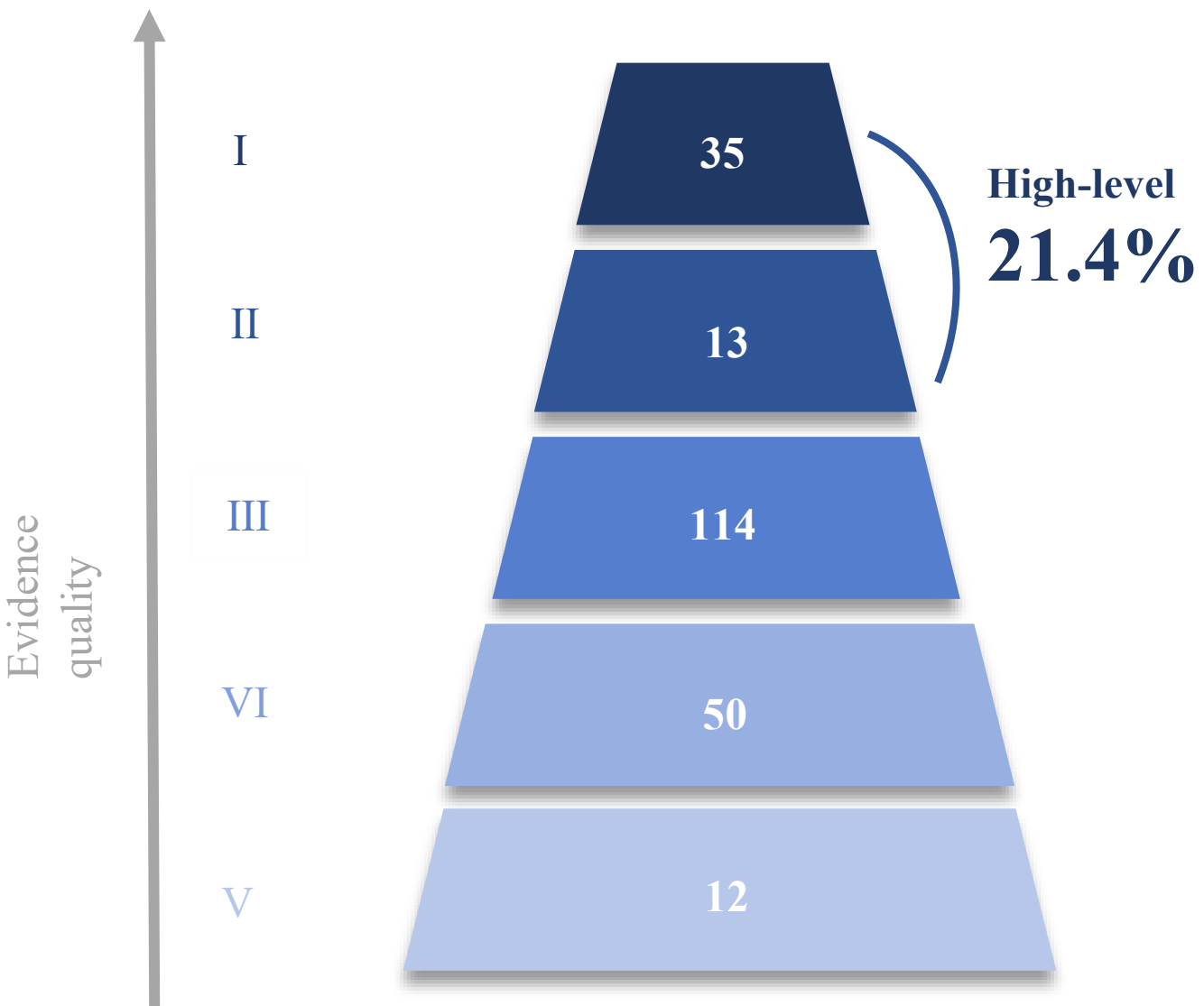

n=224 total references

B

Quantitative distribution

Proportional evidence distribution

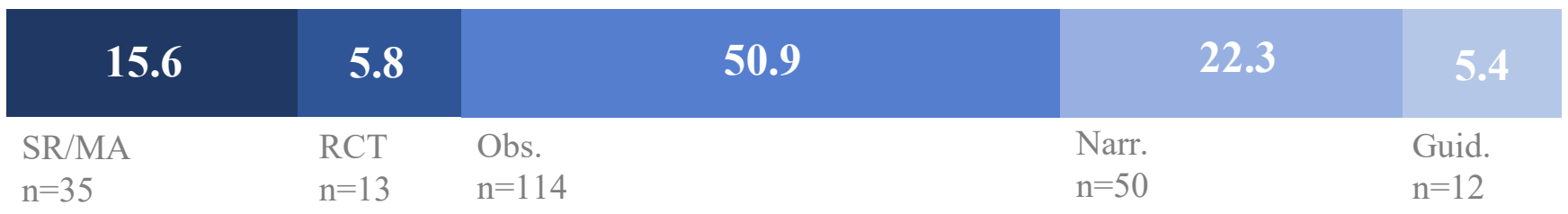

Detailed

Evidence level	Study type	n	%
I	Systematic Review /Meta-Analysis	35	**15.6**
II	Randomized Clinical Trial	13	**5.8**
III	Observational Study	114	**50.9**
VI	Narrative Review	50	**22.3**
V	Clinical Guidelines /Expert Consensus	12	**5.4**
**Total**		**224**	**100.0**

High-level evidence (I-II)
21.4% (48/224)

Primary research (II-III)
56.7% (127/224)
