# Supplementary material for: Navigating MASLD heterogeneity: from metabolic risk to precision stratification
Source: Front Med (Lausanne). 2026 Oct 30;13:1966460. doi: 10.3389/fmed.2026.1966460 (PMC13760200; doi:10.3389/fmed.2026.1966460)
Supplement: Supplementary file 2 [file Table1.docx]

| **Reference** | **Study (First Author, Year)** | **Design** | **Sample** | **Key endpoint** | **Level** | **Relevance** |
| --- | --- | --- | --- | --- | --- | --- |
| **Disease Definition and Clinical Guidelines** | | | | | | |
| (12) | Rinella M, 2023 | International consensus | _ | Subtype and classification | 5 | Nomenclature |
| (20) | Eslam M, 2021 | International consensus | _ | Subtype and management | 5 | Nomenclature/therapy |
| (22) | Cusi K, 2025 | International consensus | _ | Subtype and management | 5 | Nomenclature/ diagnosis |
| (23) | EASL-EASD-ESAO, 2024 | Clinical practice guideline | _ | MASLD management | 5 | Diagnosis /therapy |
| **Genetic risk and environmental factors** | | | | | | |
| (14) | Anstee QM, 2020 | Cohort study | 1,483 | *PNPLA3/TM6SF2/MBOAT7* SNPS | 3 | Genetic determinants |
| (46) | Liu Z, 2022 | Cohort study | 160,979 | Genetic risk score | 3 | Genetic determinants |
| (66) | Abul-Husn NS, 2018 | Cohort study | 46,544 | Protective *HSD17B13* variant | 3 | Genetic determinants |
| (69) | Diaz LA, 2025 | Cross-sectional study | 570 | Inherited genetic risk | 4 | Genetic determinants |
| (84) | Yaskolka Meir A, 2021 | Randomized controlled trial | 294 | Green-Mediterranean diet | 2 | Environmental factors |
| **Clinical phenotype and natural outcome** | | | | | | |
| (93) | Huo Z, 2026 | Cohort study | 186,221 | Liver-related clinical outcomes | 3 | Clinical heterogeneity |
| (100) | Ekstedt M, 2015 | Cohort study | 229 | Trends in mortality | 3 | Natural outcome |
| (111) | Singh S, 2015 | Meta-analysis | 411 | Fibrosis progression | 1 | Natural outcome |
| (112) | Sanyal AJ, 2021 | Cohort study | 1,773 | Liver-related clinical outcomes | 3 | Natural outcome |
| (114) | Taylor RS, 2020 | Meta-analysis | 4,428 | Fibrosis stage and mortality | 1 | Natural outcome |
| (115) | Sanyal AJ, 2022 | Cohort study | 1,135 | Cirrhosis regression | 3 | Natural outcome |
| **Non-invasive diagnosis and risk prediction** | | | | | | |
| (121) | Qu Y, 2019 | Meta-analysis | 1,100 | MRI-PDFF and steatosis | 1 | Diagnosis |
| (122) | Qadri S, 2024 | Meta-analysis | 597 | Histological steatosis and PDFF | 1 | Diagnosis |
| (123) | Rinella M, 2023 | Clinical practice guideline | - | Non-invasive evaluation | 5 | Diagnosis / therapy |
| (127) | Ferraioli G, 2024 | Clinical practice guideline | - | Ultrasound-based liver fat quantification | 5 | Diagnosis |
| (133) | Nishimura T, 2025 | Cohort study | 562 | ATI versus CAP, with MRI-PDFF | 3 | Diagnosis |
| (142) | Vali Y, 2023 | Cohort study | 1430 | ELF/FIB-4/NIS for fibrosis staging | 2 | Diagnosis |
| (145) | Serra-Burriel M, 2023 | Cohort study | 14,726 | Liver risk score | 2 | Diagnosis |
| (147) | van Kleef LA, 2024 | Cohort study | 21,797 | MAF-5 score for fibrosis risk | 3 | Diagnosis |
| **Pharmacological therapy** | | | | | | |
| (198) | Harrison SA, 2023 | Randomized controlled trial | 1,143 | Resmetirom and fibrosis/MASH | 2 | Therapy |
| (200) | Sanyal AJ, 2025 | Randomized controlled trial | 534 | Semaglutide and fibrosis/MASH | 2 | Therapy |
| (207) | Loomba R, 2025 | Randomized controlled trial | 222 | Pegzafermin and fibrosis/MASH | 2 | Therapy |
| **Bariatric surgery** | | | | | | |
| (212) | Pedersen JS, 2021 | Cohort study | 40 | Histological steatosis after RYGB/SG | 3 | Therapy |
| (213) | Verrastro O, 2023 | Randomized controlled trial | 288 | NASH resolution after RYGB/SG | 2 | Therapy |
| (214) | Pereira LH, 2025 | Meta-analysis | 322 | Advanced fibrosis outcomes of RYGB/SG | 1 | Therapy |
| (215) | Lassailly G, 2020 | Cohort study | 180 | NASH resolution and fibrosis regression | 3 | Therapy |
