# Supplementary material for: Navigating MASLD heterogeneity: from metabolic risk to precision stratification
Source: Front Med (Lausanne). 2026 Oct 30;13:1966460. doi: 10.3389/fmed.2026.1966460 (PMC13760200; doi:10.3389/fmed.2026.1966460)
Supplement: Supplementary file 3 [file Table2.docx]

| **Abbreviation** | **Definition** | **Abbreviation** | **Definition** |
| --- | --- | --- | --- |
| **2D-SWE** | Two-dimensional shear wave elastography | **LPIAT1** | Lysophosphatidylinositol acyltransferase 1 |
| **A1C** | Glycated hemoglobin | **LPCs** | Lysophosphatidylcholines |
| **AASLD** | American Association for the Study of Liver Diseases | **LSM** | Liver stiffness measurement |
| **ACC** | Acetyl-CoA carboxylase | **MAFLD** | Metabolic associated fatty liver disease |
| **ACSM** | American College of Sports Medicine | **MASH** | Metabolic dysfunction-associated steatohepatitis |
| **AHT** | Arterial hypertension | **MASLD** | Metabolic dysfunction-associated steatotic liver disease |
| **ALT** | Alanine aminotransferase | **MetALD** | Metabolic and alcohol-related liver disease |
| **Apo AI** | Apolipoprotein AI | **MGH LFS** | Massachusetts General Hospital Liver Fat Score |
| **ApoB** | Apolipoprotein B | **MICT** | Moderate-intensity continuous training |
| **AST** | Aspartate aminotransferase | **MBOAT7** | Membrane-bound O-acyltransferase domain containing 7 |
| **AUROC** | Area under the receiver operating characteristic curve | **MRI** | Magnetic resonance imaging |
| **BMI** | Body mass index | **MRI-PDFF** | Magnetic resonance imaging-proton density fat fraction |
| **CAP** | Controlled attenuation parameter | **NAFLD** | Non-alcoholic fatty liver disease |
| **CI** | Confidence interval | **NAS** | NAFLD Activity Score |
| **CK18** | Cytokeratin 18 | **NASH** | Non-alcoholic steatohepatitis |
| **CKB** | China Kadoorie Biobank | **NFS** | NAFLD fibrosis score |
| **CKD** | Chronic kidney disease | **OR** | Odds ratio |
| **CNV** | Copy number variation | **PDFF** | Proton density fat fraction |
| **CRN** | Clinical Research Network | **PI** | Phosphatidylinositol |
| **CVD** | Cardiovascular disease | **PNPLA3** | Patatin-like phospholipase domain-containing protein 3 |
| **DAGs** | Diacylglycerols | **pSWE** | Point shear wave elastography |
| **DM** | Diabetes mellitus | **RAAS** | Renin-angiotensin-aldosterone system |
| **DNL** | De novo lipogenesis | **RCTs** | Randomized controlled trials |
| **EASL** | European Association for the Study of the Liver | **ROS** | Reactive oxygen species |
| **eGFR** | Estimated glomerular filtration rate | **SAFE** | Steatosis-associated fibrosis estimator |
| **ELF** | Enhanced Liver Fibrosis | **SCDs** | Stearoyl-CoA desaturases |
| **ER** | Endoplasmic reticulum | **SGLT2** | Sodium-glucose cotransporter 2 |
| **EWAS** | Exome-wide association study | **SLD** | Steatotic liver disease |
| **FAS** | Fatty acid synthase | **SMAD3** | SMAD family member 3 |
| **FDA** | Food and Drug Administration | **SMAD4** | SMAD family member 4 |
| **FFA** | Free fatty acid | **SNS** | Sympathetic nervous system |
| **FGF21** | Fibroblast growth factor 21 | **SOS** | Swedish Obese Subjects |
| **FIB-4** | Fibrosis-4 index | **T2D** | Type 2 diabetes |
| **FIB-6** | Fibrosis-6 index | **T2DM** | Type 2 diabetes mellitus |
| **FLI** | Fatty liver index | **TFs** | Transcription factors |
| **GGT** | Gamma-glutamyl transferase | **TG** | Triglyceride |
| **GLP1-RA** | Glucagon-like peptide-1 receptor agonist | **TGs** | Triglycerides |
| **GLP1RA** | Glucagon-like peptide-1 receptor agonist | **THR-β** | Thyroid hormone receptor-β |
| **GRADE** | Grading of Recommendations Assessment, Development and Evaluation | **TM6SF2** | Transmembrane 6 superfamily member 2 |
| **GRS** | Genetic risk score | **UDCA** | Ursodeoxycholic acid |
| **GWAS** | Genome-wide association studies | **UKB** | UK Biobank |
| **HCC** | Hepatocellular carcinoma | **US** | Ultrasound |
| **HFD** | High-fat diet | **VCTE** | Vibration-controlled transient elastography |
| **HIIT** | High-intensity interval training | **VLDL** | Very low-density lipoprotein |
| **HSI** | Hepatic steatosis index | **VLDL-TG** | Very low-density lipoprotein triglyceride |
| **HSD17B13** | 17β-Hydroxysteroid dehydrogenase type 13 | **XPO4** | Exportin 4 |
| **HTN** | Hypertension | **α2m** | Alpha-2 macroglobulin |
| **IR** | Insulin resistance |  |  |
